# Supplementary material for: Journey to facility birth in Zanzibar: a questionnaire-based cohort study of patients’ perspectives on preparedness, access and quality of care
Source: BMJ Open. 2021 Feb 5;11(2):e040381. doi: 10.1136/bmjopen-2020-040381 (PMC7925926; doi:10.1136/bmjopen-2020-040381)
Supplement: Supplementary data [file bmjopen-2020-040381supp001.pdf]

Table 1: Markers for organ dysfunction, identifying cases in life-threatening condition

Type of organ dysfunction	Markers (clinical signs, laboratory markers and management actions)
**Cardiovascular**	Shock[1], use of continuous vasoactive drugs, cardiac arrest, cardio-pulmonary resuscitation, severe hypoperfusion (lactate >5 mmol/L or >45mg/dL)[2] or severe acidosis (pH <7.1)[2]
**Respiratory**	Acute cyanosis, gasping[3], severe tachypnea (respiratory rate >40bpm), severe bradypnea (respiratory rate <6bpm), severe hypoxemia (PAO2/FiO2 <200mmHg[2] or O2 saturation <90% for ≥60min) or intubation and ventilation not related to anaesthesia
**Renal**	Oliguria[4] non-responsive to fluids or diuretics, dialysis for acute renal failure[5] or severe acute azotaemia (creatinine ≥300umol/ml or ≥3.5mg/dL)
**Coagulation / haematological**	Clotting failure[6], use of continuous vasoactive drugs[7], massive transfusion of blood or red cells (≥5 units)[8] or severe acute thrombocytopenia (<50,000 platelets/ml)
**Hepatic**	Jaundice in the presence of pre-eclampsia[9], severe acute hyperbilirubinemia (bilirubin >100umol/L or >6.0mg/dL)
**Neurological**	Prolonged unconsciousness (lasting >12 hours)/coma[10], stroke[11], status epilepticus[12], uncontrollable fits/total paralysis
**Uterine**	Hysterectomy following haemorrhage or infection

1) Shock is a persistent severe hypotension, defined as a systolic blood pressure <90 mmHg for ≥60 minutes with a pulse rate at least 120 despite aggressive fluid replacement (>2l)
2) Laboratory test or management intervention that is not available at MMH
3) Gasping is a terminal respiratory pattern and the breath is convulsively and audibly caught
4) Oliguria is defined as a urinary output <30 ml/hr for 4 hours or <400 ml/24 hr
5) Dialysis services have been available in MMH since 26 May 2017
6) Clotting failure can be assessed by the bedside clotting test or absence of clotting from the intravenous site after 7–10 minutes
7) For instance, continuous use of any dose of dopamine, epinephrine or norepinephrine
8) In MMH extended to include all types of blood products and cases in which 5 or more units were requested but not given due to shortage
9) Pre-eclampsia is defined as the presence of hypertension associated with proteinuria. Hypertension is defined as a blood pressure of at least 140 mmHg (systolic) or at least 90 mmHg (diastolic) on at least two occasions and at least 4–6 h apart after the 20th week of gestation in women known to be normotensive beforehand. In MMH, proteinuria is defined as ≥2+ protein on dipstick.
10) Loss of consciousness is a profound alteration of mental state that involves complete or near-complete lack of responsiveness to external stimuli. It is defined as a Coma Glasgow Scale <10 (moderate or severe coma).
11) Stroke is a neurological deficit of cerebrovascular cause that persists beyond 24 hours or is interrupted by death within 24 hours
12) Condition in which the brain is in a state of continuous seizure

Table 2: Interview outline

PATIENT INTERVIEW – BEFORE DISCHARGE (English version)			**NO.:**
**Topic**	**Questions**		
**1) Patient characteristics**	Age:	Level of education (primary, secondary, tertiary):	
	Gravidity/Parity:	Marital status (first-, second-, third- or fourth wife, divorced, widow, single):	
	Number of living children:	Perceived wealth:<br>Very poor – Poor – Average – Rich – Very Rich	
	Number of children of school-going age attending school:	Occupation:<br>Paid work – Self-employed – Non-paid work – Student – Housework – Unemployed for health reason – Unemployed for other reason – Other, …………………	
	Smoking?	Alcohol?	Drugs?
**2) Family Planning**	Pregnancy planned?	If unplanned, wanted?	Did you have access to contraceptives?

		**1**	**2**	**3**	**4**	**5**	**na**
**3) Antenatal Care (ANC)**	**How did you experience the access of ANC?** Scale: 1=impossible, 2=difficult, 3=not simple, not difficult, 4=simple, 5= very simple, na=not applicable						
	**How do you feel about your overall experience in ANC?** Scale: 1=very dissatisfied, 2=dissatisfied, 3=neither satisfied, nor dissatisfied, 4=satisfied, 5= very satisfied, na=not applicable						

**3) Antenatal Care (ANC)**	**Did you consult others for treatment during your pregnancy such as traditional birth attendants or others?**	
	a. No	
	b. Yes, traditional birth attendant	
	c. Yes, other	
	**Comments:**	
**4) Access to Health Facility**	**Where did you plan to deliver?**	
	a. Home	
	b. Health center	
	c. Other hospital	
	d. MMH	
	**What was the <u>main</u> motivation for delivering there?**	
	**Who accompanied you to the health facility and took care of you during hospitalization?**	
	a. Female family member	
	b. Husband	
	c. Friend	
	d. Other	
	**Who took care of your children during your hospitalization?**	
	a. Female family member	
	b. Husband	
	c. Friend	
	d. Other	

		1	2	3	4	5	na
	**How did you experience the access to the health facility?** Scale: 1=very poor, 2=poor, 3=not good, not poor, 4=good, 5= very good, na=not applicable						
	**If you encountered problems, what kind of problems?**						
	a. Financial						
	b. Referral						
	c. Logistic						
	d. No problems						
	**How did you come here and how long did that take?**						
	**Comments:**						
**5) Perception on Development of Disease/ Complications**	**How much time passed between when you started feeling less well and you sought medical help?**						
	**How much time passed between when you arrived in the hospital/you started feeling less well in the hospital and the moment a health worker came to see/question/examine you?**						
	**Did you recognise that you started to feel worse? If so, how did you feel and what did you think?**						
		1	2	3	4	5	na
	**Did you have knowledge about your disease/ complication(s) before you suffered from it yourself? If so, how?** Scale: 1=not at all, 2= little, 3= moderate amount, 4=good knowledge, 5=extreme good knowledge, na=not applicable						
	**Comments**						
		1	2	3	4	5	na
**6) Perceived Quality of Care during Hospitalization**	**Did you feel treated with respect?** Scale: 1=not at all, 2=a little, 3=a moderate amount, 4=very much, 5=extremely, na=not applicable						
	**Did you feel informed about treatments/ complications?** Scale: 1=not at all, 2=a little, 3=a moderate amount, 4=very much, 5=extremely, na=not applicable						
	**Did you feel you had an informed choice in the services you received?** Scale: 1=not at all, 2=a little, 3=a moderate amount, 4=very much, 5=extremely, na=not applicable						
	**Did you feel you had privacy?** Scale: 1=not at all, 2=a little, 3=a moderate amount, 4=very much, 5=extremely, na=not applicable						
	**Were you able to receive emotional support from relatives/friends?** Scale: 1=not at all, 2=a little, 3=a moderate amount, 4=very much, 5=extremely, na=not applicable						
	**Did you feel you were provided with emotional support by the staff?** Scale: 1=not at all, 2=a little, 3=a moderate amount, 4=very much, 5=extremely, na=not applicable						

	**Did you feel that the staff was motivated and available?** Scale: 1=not at all, 2=a little, 3=a moderate amount, 4=very much, 5=extremely, na=not applicable						
	**Did you feel safe in the hospital?** Scale: 1=not at all, 2=a little, 3=a moderate amount, 4=very much, 5=extremely, na=not applicable						
	**Did you feel the health workers took time for you?** Scale: 1=not at all, 2=a little, 3=a moderate amount, 4=very much, 5=extremely, na=not applicable						
	**Did you experience financial barriers to getting the right treatment?** Scale: 1=extremely, 2=very much, 3=a moderate amount, 4=a little, 5=not at all, na=not applicable						
	**Did you think the hospital had sufficient supplies to care for you? (Sanitation, medicine, equipment)** Scale: 1=not at all, 2=a little, 3=a moderate amount, 4=very much, 5=extremely, na=not applicable						
	**How do you feel about the way your baby was treated?** Scale: 1=very dissatisfied, 2=dissatisfied, 3=neither satisfied, nor dissatisfied, 4=satisfied, 5= very satisfied, na=not applicable						
	**How do you feel about the overall experience of your admission?** Scale: 1=very dissatisfied, 2=dissatisfied, 3=neither satisfied, nor dissatisfied, 4=satisfied, 5= very satisfied, na=not applicable						
	If your baby died:						
		**1**	**2**	**3**	**4**	**5**	**na**
	**How do you feel about the way you were informed on the death of your baby?** Scale: 1=very dissatisfied, 2=dissatisfied, 3=neither satisfied, nor dissatisfied, 4=satisfied, 5= very satisfied, na=not applicable						
	**How do you feel about the way you, your baby and the baby's father were treated after your baby's death?** Scale: 1=very dissatisfied, 2=dissatisfied, 3=neither satisfied, nor dissatisfied, 4=satisfied, 5= very satisfied, na=not applicable						
	**Comments:**						
	**SCALE** 1=an extreme impact, 2= a high impact, 3=a moderate impact, 4= little impact, 5= no impact at all, na=not applicable	**1**	**2**	**3**	**4**	**5**	**na**
**7) Perspective on Impact of Hospitalization**	**Impact on your position within your close family**						
	**Impact on your position within extended family/community**						
	**Financial and economic impact of hospitalization**						
	**Impact on educational prospects your children**						
	**Comments:**						
**8) State upon Discharge**	**Do you feel recovered at discharge?** Yes/No						
	**Do you feel able to resume your daily activities?** Yes/No						

**9) Recommend**	**Would you recommend your sister to deliver in the same place?** Yes/No
**10) Suggestions**	**Do you have any suggestions to improve the care for you and your baby in the future? What would you like to see different?**
