# Supplementary material for: Journey to facility birth in Zanzibar: a questionnaire-based cohort study of patients’ perspectives on preparedness, access and quality of care
Source: BMJ Open. 2021 Feb 5;11(2):e040381. doi: 10.1136/bmjopen-2020-040381 (PMC7925926; doi:10.1136/bmjopen-2020-040381)
Supplement: Author's manuscript [file bmjopen-2020-040381.draft_revisions.pdf]

BMJ Open is committed to open peer review. As part of this commitment we make the peer review history of every article we publish publicly available.

When an article is published we post the peer reviewers' comments and the authors' responses online. We also post the versions of the paper that were used during peer review. These are the versions that the peer review comments apply to.

The versions of the paper that follow are the versions that were submitted during the peer review process. They are not the versions of record or the final published versions. They should not be cited or distributed as the published version of this manuscript.

BMJ Open is an open access journal and the full, final, typeset and author-corrected version of record of the manuscript is available on our site with no access controls, subscription charges or pay-per-view fees (http://bmjopen.bmj.com).

If you have any questions on BMJ Open's open peer review process please email info.bmjopen@bmj.com

BMJ Open

The journey to facility birth in Zanzibar: patients' perspectives on preparedness, access and quality of care

Journal:	*BMJ Open*
Manuscript ID	bmjopen-2020-040381
Article Type:	Original research
Date Submitted by the Author:	23-May-2020

Complete List of Authors:	Herklots, Tanneke; Erasmus MC, Obstetrics & Gynaecology<br>D'haene, Lara; Hospital Doctor Jose Molina Orosa, Emergency Medicine<br>Mbarouk, Khairat; Mnazi Mmoja Referral Hospital, Obstetrics & Gynaecology<br>Rajhy, Mubina; Mnazi Mmoja Referral Hospital, Obstetrics & Gynaecology<br>Couperus, Simone; University of Utrecht Faculty of Medicine, Obstetrics &Gynaecology<br>Meguid, Tarek ; Village Health Works<br>Franx, Arie; Erasmus MC, Obstetrics & Gynaecology<br>Koster, Maria PH; Erasmus MC, Obstetrics & Gynaecology<br>Jacod, Benoit; Onze Lieve Vrouwe Gasthuis, Obstetrics & Gynaecology

Keywords:	Maternal medicine < OBSTETRICS, Quality in health care < HEALTH SERVICES ADMINISTRATION & MANAGEMENT, Organisation of health services < HEALTH SERVICES ADMINISTRATION & MANAGEMENT, REPRODUCTIVE MEDICINE

SCHOLARONE™
Manuscripts

I, the Submitting Author has the right to grant and does grant on behalf of all authors of the Work (as defined in the below author licence), an exclusive licence and/or a non-exclusive licence for contributions from authors who are: i) UK Crown employees; ii) where BMJ has agreed a CC-BY licence shall apply, and/or iii) in accordance with the terms applicable for US Federal Government officers or employees acting as part of their official duties; on a worldwide, perpetual, irrevocable, royalty-free basis to BMJ Publishing Group Ltd ("BMJ") its licensees and where the relevant Journal is co-owned by BMJ to the co-owners of the Journal, to publish the Work in this journal and any other BMJ products and to exploit all rights, as set out in our licence.

The Submitting Author accepts and understands that any supply made under these terms is made by BMJ to the Submitting Author unless you are acting as an employee on behalf of your employer or a postgraduate student of an affiliated institution which is paying any applicable article publishing charge ("APC") for Open Access articles. Where the Submitting Author wishes to make the Work available on an Open Access basis (and intends to pay the relevant APC), the terms of reuse of such Open Access shall be governed by a Creative Commons licence – details of these licences and which Creative Commons licence will apply to this Work are set out in our licence referred to above.

Other than as permitted in any relevant BMJ Author's Self Archiving Policies, I confirm this Work has not been accepted for publication elsewhere, is not being considered for publication elsewhere and does not duplicate material already published. I confirm all authors consent to publication of this Work and authorise the granting of this licence.

For peer review only

The journey to facility birth in Zanzibar: patients' perspectives on preparedness, access and quality of care

Tanneke Herklots[1*], Lara D'haene[2], Khairat Mbarouk Said[3], Mubina Rajhy[3], Simone Couperus[4], Tarek Meguid[5], Arie Franx[1], Maria PH Koster[1], Benoit Jacod [6]

1. Department of Obstetrics & Gynaecology, Erasmus MC, University Medical Centre Rotterdam, Rotterdam, The Netherlands
2. Department of Emergency Medicine, Hospital Doctor Jose Molina Orosa, Lanzarote, Spain
3. Department of Obstetrics & Gynaecology, Mnazi Mmoja Hospital, Zanzibar, United Republic of Tanzania
4. Department of Obstetrics & Gynaecology, University Medical Centre Utrecht, Utrecht, The Netherlands
5. Village Health Works, Kigutu, Burundi
6. Department of Obstetrics & Gynaecology, Onze Lieve Vrouw Gasthuis, Amsterdam, The Netherlands

*) Corresponding author: full name: Tanneke Herklots; postal address: Kromme Slagen 11, 4823 LL, Breda, The Netherlands; e-mail: tannekeherklots@gmail.com

Word count abstract: 300

Word count full text: 3262

For peer review only

ABSTRACT

Introduction

Tackling substandard childbirth and maternity care in health facilities requires engaging the women's perspectives in designing strategies to improve outcomes. This study aims to provide insights in patients' perspectives on preparedness, access and quality of care in Mnazi Mmoja Hospital, Zanzibar, Tanzania.

Methods

In a prospective cohort study from April 2017 until December 2018, we performed semi-structured interviews with women who experienced maternal near-miss complications and matched control participants. These focussed on sociodemographic and obstetric characteristics, perceived accessibility to facility care and perceived quality of facility care with 15 domains, scored on a one-to-five scale. Participants' comments and answers to open questions were employed to illustrate quantitative outcomes. Zanzibar's Medical Research and Ethics Committee approved the study (ZAMREC/0002/JUN/17).

Results

We included 174 patients who experienced maternal near-miss complications and 151 controls. Compared to controls, patients in the near-miss group had less formal education ($p=0.049$), perceived their wealth as poor ($p=0.002$), and more often had a stillbirth ($p<0.001$). For many, there was a delay in deciding to seek care. More than controls, women in the near-miss group experienced barriers in reaching care ($p=0.049$), often of financial nature (13.8% vs. 4.0%). Quality of care was perceived as high, with means above three out of five, in 14 out of 15 domains. One fifth had an overall suboptimal experience, mostly regarding informed choice and supplies availability. Critical comments were expressed by a minority of participants.

Conclusion

Most patients promptly seek, access and receive maternity care in Zanzibar's referral hospital. A minority experiences barriers, mostly financial, in reaching care, and more so among women

For peer review only

experiencing maternal near-miss complications. Quality of facility care is generally highly rated. However, a group does report insightful critical perceptions. For patient-centered and setting-specific maternal health research aiming to improve maternal well-being, we suggest combining quantitative and qualitative methodologies.

Key words: maternal health, near-miss, patient perspective, access to care, quality of care, low-resource setting

STRENGTHS AND LIMITATIONS OF THIS STUDY

- The study involved a wide range and large number of maternal patients in Zanzibar's main hospital
- Because we could not include women who had not reached the hospital, nor patients who were not in possession of a mobile phone, the study had a selection bias potentially at the cost of those suffering more from poverty
- There might have been a certain level of information bias because the (majority of) interviews was conducted in the hospital, potentially discouraging people to criticize the facility they were at
- The given comments reached outcome saturation, for which we trust them to experienced reality, thus being valid starting points for healthcare improvement to provide to policy makers
- This study displays how facility maternity care is perceived by the patients themselves, and does so in an extensive, profound and both quantitative and qualitative manner

For peer review only

INTRODUCTION

Low-income countries carry a disproportionately large burden of maternal morbidity and mortality. Efforts have gone into ensuring availability of essential interventions, promoting facility deliveries and skilled birth attendance,[1]. Unfortunately, despite a widespread increase in facility deliveries, the improvement in pregnancy outcomes has been slower than expected. This can partly be explained by of limited preparedness to seek care on time and suboptimal access to care, but there seems to be a causal role for the low quality of care provided in many facilities,[2]. Low quality of care negatively affects the willingness to seek facility care, either in a future pregnancy or of other pregnant women in the community. Poor quality of care is not limited to but includes maltreatment during childbirth, which has been found to be common in many low-income settings,[3-5]. Tackling substandard maternity care requires incorporating the perspectives of women in designing strategies to improve maternal and child outcomes.

Our aim was to gain insight into preparedness for, access to and quality of care from the patients' perspectives on their journey to childbirth in Zanzibar's main hospital, Mnazi Mmoja Hospital (MMH). In Zanzibar, Tanzania, around half of the deliveries take place at home and half in a health facility,[6]. Of the latter, around 30% take place in the region's main public referral health facility: MMH. The maternal mortality rate (MMR) at MMH is around 400 per 100,000 livebirths,[7]. Multiple quality of care assessments have been performed at Tanzanian health care facilities guided by the World Health Organization's (WHO) Standard for Care,[8]. In 2012, a large unmet need for basic and comprehensive emergency obstetric and neonatal care was found throughout Zanzibar, including shortages of staff and supplies, and an insufficient geographical access,[9]. In 2016, an assessment in Zanzibar showed that five main governmental hospitals, including MMH, were overall not conducive for high-quality maternal and child health care provision (Meguid T, Mrisho Haji F, Herklots T, Rajhy M, Kassim NI. Conduciveness for High Quality of Maternal- and Child Health Care in Hospitals in Zanzibar. 2016). In 2017, an assessment of 37 maternity units in Zanzibar showed how more than half of those had challenges in providing hand and surface hygiene,[10].

For peer review only

In this study, we assessed the experiences of two groups of women in pregnancy and childbirth, those with and without severe, life-threatening complications. This allowed us to take into account the effect of pregnancy outcomes on patients' perceptions.

METHODS

From April 2017 until December 2018, we conducted a prospective cohort study at the department of Obstetrics & Gynaecology of MMH. We performed semi-structured interviews with women who experienced a maternal near-miss (MNM) and control participants. MNM were identified during the department's twice daily meetings by a locally adapted and validated version of the WHO near-miss approach (for criteria see Supplementary Files, Table 1),[7]. Controls, matched on a 1:1 ratio, had uncomplicated or mildly complicated pregnancy and a similar date of admission, similar mode of delivery and similar gestational age as MNM.

This research was approved by Zanzibar's Medical Research and Ethics Committee (ZAMREC/0002/JUN/17) and conducted in line with the principles of the Declaration of Helsinki,[11]. In addition to providing informed consent, inclusion criteria were age of 18 years or above, no severe pre-diagnosed psychiatric disorders, residence on Unguja (Zanzibar's main island on which MMH is located) and to be reachable by mobile phone. Informed consent of all participants was obtained in writing or verbally, in case of illiteracy. An interview was performed before discharge or, in case of logistic restrictions, within one week of discharge at the participant's home. The interviews were performed by a researcher and a mediator/translator, both not involved in the clinical care of the woman. The interview comprised the following: sociodemographic circumstances (age, self-reported level of formal education, type of occupation, marital status, perceived wealth), obstetric characteristics (parity – with a grand multiparity defined as a parity above 4, singleton or multiple pregnancy, gestational age, planned location for delivery and pregnancy outcome being abortion, live birth or still birth), utilization and opinion of antenatal care (ANC) services (not included in this

For peer review only

publication), perception on the accessibility of facility care and perception on the quality of facility care (see Supplementary Files, Table 2, for the interview outline).

SPSS Statistics (version 25) and OpenEpi (version 3.01) were used for statistical analysis. Numeric outcomes were categorized similarly as in previous studies in this setting: age younger than 20 years, 20 to 35 years and older than 35 years; and parity zero, one to four or higher than four,[7,12]. Baseline characteristics were compared using Pearson's chi-square tests and, in case of small sample sizes, Fisher's exact test. Regarding quality of care, the interview outcomes were on a five-point scale, from which means per question were calculated and compared between MNM and controls using Mann-Whitney-U-tests. Suboptimal care was defined as a score of 3 or lower. Proportions of participants grading the assessed element as suboptimal were reflected in percentages. Odds ratios (OR) with 95% confidence intervals (CI) were calculated to compare between MNM and controls, to assess if having had a pregnancy with a life-threatening complication affects quality of care perception. We observed a discrepancy between the fact that the majority of patients perceived quality of care as good or very good and their given comments, most often of critical nature. To investigate this discrepancy further, we compared the frequency of negative comments in participants having scored as positive or as suboptimal on the domain to which the comments pertain. We deemed the number of participants giving comments to be sufficiently high to perform this analysis on four domains: information, informed choice, respect and privacy.

A p-value below 0.05 was considered statistically significant. Quotations taken from participants' comments and answers to open questions were employed to illustrate the quantitative outcomes.

RESULTS

During the study period, 269 patients were identified as MNM, of whom 174 (64.7%) were interviewed. Reasons for no study participation were patients' personal reasons, meeting one

For peer review only

of the exclusion criteria or an unexpected early discharge home. A total of 151 control participants were interviewed.

Participants' characteristics

Sociodemographic and obstetric baseline characteristics are displayed in Table 1. MNM and controls were comparable at baseline regarding age, occupation and marital status. MNM were significantly more likely to have received primary education only (29.9% vs. 19.2%, p=0.05), more often perceived their own wealth as very poor or poor (19.0% vs. 7.3%, p=0.002), were less often nulliparous (32.2% vs. 50.3%, p=0.002), and more often grand multiparous (13.9% vs. 6.0%, p=0.002). Of the MNM pregnancies, 42.9% ended in a stillbirth compared to 5.4% of control pregnancies (p<0.001). Nearly two-thirds of all participants had initially planned to deliver at MMH, controls significantly more so than MNM (total 66.5%; 58.6% MNM vs. 75.5% controls, p=0.004).

Table 1: Sociodemographic and obstetric baseline characteristics

	**Total N (%)**	**MNM N (%)**	**Controls N (%)**	**p-value**
**Total**	325 (100)	174 (53.5)	151 (46.5)	
**Age**				0.25
Data available[1]	324 (99.7)	174 (100)	150 (99.3)	
<20 years	16 (4.9)	11 (6.3)	5 (3.3)	
20-35 years	267 (82.4)	138 (79.3)	129 (86.0)	
>35 years	41 (12.7)	25 (14.4)	16 (10.7)	
**Parity**				**0.002**
Data available[1]	324 (99.7)	173 (99.4)	151 (100)	
0 (nulliparous)	132 (40.7)	56 (32.4)	76 (50.3)	
1-4 (multiparous)	159 (49.1)	93 (53.8)	66 (43.7)	
5 or more (grand multiparous)	33 (10.2)	24 (13.9)	9 (6.0)	
**Formal education level**				**0.049**
No formal education	24 (7.4)	15 (8.6)	9 (6.0)	
Primary	81 (24.0)	52 (29.9)	29 (19.2)	
Secondary	194 (59.7)	97 (55.7)	97 (64.2)	
Higher	26 (8.0)	10 (5.7)	16 (10.6)	
**Occupation**				0.80
Paid work or self-employed	148 (45.5)	76 (43.7)	72 (47.7)	

For peer review only

Housework	163 (50.2)	91 (52.3)	72 (47.7)	
Non-paid work or student	9 (2.8)	5 (2.9)	4 (2.6)	
Unemployed	5 (1.5)	2 (1.1)	3 (2.0)	
**Marital status**				0.21
Married	299 (92.0)	157 (90.2)	142 (94.0)	
Unmarried	26 (8.0)	17 (9.8)	9 (6.0)	
**Perceived wealth**				**0.002**
Very poor or poor	44 (13.5)	33 (19.0)	11 (7.3)	
Average	280 (86.2)	140 (80.5)	140 (92.7)	
Rich or very rich	1 (0.3)	1 (0.6)	0 (0.0)	
**Singleton/multiple pregnancy[2]**				**0.03**
Data available	261 (80.3)	144 (82.8)	117 (77.5)	
Singleton	242 (92.7)	129 (89.6)	113 (96.6)	
Multiple	19 (7.3)	15 (10.4)	4 (3.4)	
**Planned location for delivery**				**0.004**
MMH	216 (66.5)	102 (58.6)	114 (75.5)	
Other hospital	97 (29.8)	63 (36.2)	34 (22.5)	
Health centre	8 (2.5)	6 (3.4)	2 (1.3)	
Home	4 (1.2)	3 (1.7)	1 (0.7)	
**Pregnancy outcome**				**<0.001**
Data available[1]	319 (98.2)	170 (97.7)	149 (98.7)	
Ending before 20 weeks	43 (13.5)	25 (14.7)	18 (12.1)	
Live birth	191 (59.9)	69 (40.6)	122 (81.9)	
Stillbirth	81 (25.4)	73 (42.9)	8 (5.4)	
Still pregnant at discharge	4 (1.3)	3 (1.8)	1 (0.7)	

1) In case of missing data, the table shows the available data per category in the first row; the consequent proportions are calculated with the available data as denominator
2) This excludes participants who were admitted with a gestational age below 20 weeks, including pregnancies which ended in abortion and ectopic pregnancies

Access to care

Motivations for choosing a delivery location did not differ between MNM and controls and were most often because of previous experience and expectations (36.6%) or medical indication (28.0%).

"I didn't decide yet in which hospital but I don't want to deliver at home because you can have a problem and not be able to solve it" – MNM participant, 25 years old, first pregnancy

For peer review only

The majority of MNM (59.2%) initiated their journey to MMH because they recognised that their condition was deteriorating, whilst less than a quarter (23.0%) of them said they were unaware.

"I recognized it was not good. I thought I needed to have an operation because that is what happened to my sister-in-law." – control participant, 36 years old, fourth pregnancy

Table 2 displays the various elements on access to care investigated. Over half of the MNM (55.6%) and the controls (66.1%) said to have taken more than two hours before they decided to seek medical care after starting to feel sick or labour started (p=0.18). After that, it took 36.2% of MNM and 29.1% of controls longer than 30 minutes to reach a health facility (p=0.10). Once arrived, the vast majority was attended to promptly, but some had waited longer than three hours, and MNM significantly more often than controls (9.3% vs. 5.4%, p=0.04).

Close to one fifth of all interviewees (18.6%) experienced the access to care as suboptimal, with a significant higher number of MNM than controls reporting to have encountered problems of financial, logistical or referral-related nature (22.4% MNM vs 13.9% controls, p=0.049). Many interviewees shared that they had struggled with finding and paying petrol, a borrowed car or public transportation.

"I had to wait and find a bus for six hours. There should be transport available" – MNM participant, 19 years old, second pregnancy

"I came by ambulance from [my area] and paid 10,000TSH (equivalent to 5 USD, authors). I find it not fair of them that I had to pay. What if I had no money?" – MNM participant (area of residence to MMH around a 20-minutes-drive, 8-9km), 40 years old, sixth pregnancy

For peer review only

"Because my husband was not able to buy petrol for our car, I took a dala dala (public mini bus, authors) for three hours from [my area] to MMH." – MNM participant (area of residence to MMH around one and a half hour-drive by car, 40km), 38 years old, eight pregnancy

Table 2: Experience of accessibility of care

	**Total N (%)**	**MNM N (%)**	**Controls N (%)**	**p-value[1]**
**Total**	325 (100.0)	174 (53.5)	151 (46.5)	
**Sought care X time after complaints started *(delay 1)***				0.18
Data available[2]	284 (87.4)	151 (86.8)	133 (88.1)	
Immediately	60 (21.1)	40 (26.5)	20 (15.0)	
<2 hours	35 (12.3)	19 (12.6)	16 (12.0)	
2-8 hours	96 (33.8)	45 (29.8)	51 (38.3)	
>8 hours	76 (26.8)	39 (25.8)	37 (27.8)	
Not applicable	17 (6.0)	8 (5.3)	9 (6.8)	
**Reached care X time after start of journey *(delay 2)***				0.10
Data available[2]	261 (80.3)	129 (74.1)	132 (87.4)	
<15min	70 (25.7)	36 (26.1)	34 (25.4)	
15-30min	102 (37.5)	43 (31.2)	59 (44.0)	
30-60min	49 (18.0)	24 (17.4)	25 (18.7)	
>60min	40 (14.7)	26 (18.8)	14 (10.4)	
**Received care X time after arrival in health facility *(delay 3)***				**0.04**
Data available[2]	304	158	146	
Immediately	251 (78.7)	122 (71.3)	129 (87.2)	
<3hrs	29 (9.1)	20 (11.7)	9 (6.1)	
>3hrs	24 (7.5)	16 (9.3)	8 (5.4)	
**Experienced access to care**				0.48[3]
Data available[2]	322	171	151	
Very poor	2 (0.6)	2 (1.2)	-	
Poor	10 (3.1)	5 (2.9)	5 (3.3)	
Not poor, not good	48 (14.9)	28 (16.4)	20 (13.2)	
Good	59 (18.3)	35 (20.5)	24 (15.9)	
Very good	203 (63.0)	101 (59.1)	102 (67.5)	
**Encountered problems**				**0.049[4]**
None	265 (81.5)	135 (77.6)	130 (86.1)	
Any	60 (18.5)	39 (22.4)	21 (13.9)	
Financial	30 (9.2)	24 (13.8)	6 (4.0)	
Referral from one health facility to	10 (3.1)	6 (3.4)	4 (2.6)	

For peer review only

another				
Logistics	20 (6.2)	9 (5.2)	11 (7.3)	

1) p-values are calculated with Chi-Square tests excluded the row of 'data available'; 2) the proportion of data available is calculated, the proportion of the following subcategories are calculated with the available data as the denominator; 3) this p-value has been calculated with combining the subcategories 'very poor' and 'poor'; 4) this p-value has been calculated by comparing the categories 'none' and 'any'

Quality of care

Table 3 displays the means of the outcomes per interview question reflecting quality of care. Most of these given scores are high, i.e. above four. Lowest scores regard having an informed choice (total group 2.67, MNM 2.49 and controls 2.86) and being informed (total group 3.87, MNM 3.61 and controls 4.17), including being informed about the death of their baby (total group 3.90, MNM 3.89 and controls 4.00). Mean differences between MNM and controls were statistically significant for some topics, such as having felt informed and perception of motivation of staff, but reflect small differences. One participant summarized her experience as follows:

"I am very satisfied because I am alive" – MNM participant, 28 years old, first pregnancy

Table 3: Means on a five-point scale (1=very poor, 2=poor, 3=not poor, not good, 4=good, 5=very good) and mean differences

	**Total**	**MNM**	**Controls**	
	**Mean (SD)**	**Mean (SD)**	**Mean (SD)**	**Mean difference (95% CI)**
Did you feel treated with respect?	4.54 (0.80)	4.50 (0.85)	4.58 (0.74)	-0.07 (-0.25–0.10)
Did you feel informed about treatments/ complications?	3.87 (1.54)	3.61 (1.62)	4.17 (1.40)	***-0.55 (-0.89–0.22)***
Did you feel you had an informed choice in the services you received?	2.67 (1.80)	2.49 (1.76)	2.86 (1.84)	-0.37 (-0.76–0.03)
Did you feel you had privacy?	4.47 (0.96)	4.36 (1.12)	4.59 (0.72)	***-0.23 (-0.44–0.02)***
Were you able to receive emotional support from relatives/friends?	4.47 (0.97)	4.51 (0.91)	4.42 (1.03)	0.09 (-0.12–0.30)
Did you feel you were provided with emotional support by the staff?	4.27 (1.18)	4.23 (1.20)	4.32 (1.16)	-0.09 (-0.35–0.17)
Did you feel that the staff was motivated and available?	4.41 (0.88)	4.32 (0.95)	4.52 (0.78)	***-0.20 (-0.40–0.01)***

For peer review only

Did you feel safe in the hospital?	4.65 (0.67)	4.63 (0.69)	4.66 (0.64)	-0.03 (-0.18–0.11)
Did you feel the health workers took the time for you?	4.51 (0.83)	4.47 (0.87)	4.56 (0.78)	-0.08 (-0.26–0.10)
Did you experience financial barriers to getting the right treatment?	4.59 (0.88)	4.47 (1.00)	4.74 (0.70)	***-0.27 (-0.46–0.08)***
Did you think the hospital had sufficient supplies to care for you? (Sanitation, medicine, equipment)	4.01 (0.99)	3.91 (0.97)	4.10 (1.02)	-0.17 (-0.39–0.05)
How do you feel about the way your baby was treated?	4.67 (0.77)	4.64 (0.87)	4.69 (0.70)	-0.05 (-0.28–0.18)
How do you feel about the overall experience of your admission?	4.33 (0.90)	4.24 (0.93)	4.42 (0.86)	-0.19 (-0.38–0.01)
In case baby died: how do you feel about the way you were informed on the death of your baby?	3.90 (1.54)	3.89 (1.56)	4.00 (1.48)	-0.11 (-1.08–0.85)
In case baby died: how do you feel about the way you, your baby and the baby's father were treated after your baby's death?	4.16 (1.39)	4.31 (1.26)	3.27 (1.85)	***1.04 (0.16–1.91)***
Would you recommend your sister/friend to deliver in the same place?	**Total N (%)**	**MNM N (%)**	**Controls N (%)**	
Yes	309 (95.1)	164 (94.3)	145 (96.0)	0.85 (0.57–1.26)
No	16 (4.9)	10 (5.7)	6 (4.0)	

As displayed in Table 4, a total of 70 interviewees (21.5%), comprising 43 MNM (24.7%), and 27 controls (17.9%), perceived the overall experience of the care during their admission as suboptimal. Most participants rated informed choice poorest, being scored as suboptimal by 57.5% of all participants. This is followed by sufficiency of supplies (28.3%), being informed in general (28.3%), and being informed in case of death of their baby (25.9%).

"If I had money I would go to a private hospital. The first doctor after the operation didn't tell me my baby died. … I felt ignored. …They didn't tell me anything. I am heartbroken here." – MNM participant, 31 years old, second pregnancy

Table 4: Suboptimal scores on quality of care reflecting absolute numbers and the proportion of the group of participants who answered with 1, 2 or 3. Odds ratios (OR) calculated for MNM vs. controls.

	Total, N(%)	MNM, N(%)	Control, N(%)	OR (95% CI)
**Total**	325 (100.0)	174 (53.5)	151 (46.5)	-
**Overall experience**	70 (21.5)	43 (24.7)	27 (17.9)	1.51 (0.88-2.59)

For peer review only

**Informed choice**	187 (57.5)	108 (62.1)	79 (52.3)	1.49 (0.96-2.32)
**Sufficient supplies**	104 (32.0)	61 (35.1)	43 (28.5)	1.36 (0.85-2.17)
**Information**	92 (28.3)	61 (35.1)	31 (20.5)	***2.09 (1.26-3.46)***
**Emtional support staff**	66 (20.3)	37 (21.3)	29 (19.2)	1.14 (0.66-1.96)
**Availability and motivation of staff**	57 (17.5)	38 (21.8)	19 (12.6)	***1.94 (1.07-3.54)***
**Time for patient**	43 (13.2)	22 (12.6)	21 (13.9)	0.90 (0.47-1.70)
**Financial barriers**	42 (12.9)	31 (17.8)	11 (7.3)	***2.76 (1.34-5.70)***
**Privacy**	41 (12.6)	29 (16.7)	12 (7.9)	***2.32 (1.14-4.72)***
**Emotional support family**	39 (12.0)	20 (11.5)	19 (12.6)	0.90 (0.46-1.76)
**Respect**	36 (11.1)	22 (12.6)	14 (9.3)	1.42 (0.70-2.88)
**Feeling of safety**	18 (5.5)	10 (5.7)	8 (5.3)	1.09 (0.42-2.84)
**Treatment of the baby**	10 (3.1)	4 (5.8)	6 (4.9)	0.57 (0.16-2.05)
		**Total N=73**	**Total N=12**	
**In case baby died: information**	22 (6.7)	20 (27.4)	2 (16.7)	***9.68 (2.22-42.11)***
**In case baby died: supportive treatment**	13 (4.0)	9 (12.3)	4 (33.3)	2.01 (0.60-6.65)

The odds of experiencing care as suboptimal were significantly higher for MNM concerning financial barriers in receiving high quality care (OR 2.76, 95% CI 1.34-5.70), privacy (OR 2.32, 95% CI 1.14-4.72), information provision (OR 2.09, 95% CI 1.26-3.46), and availability and motivation of staff (OR 1.94, 95% CI 1.07-3.54).

“They didn't tell me anything. I had no choice. I didn't feel extremely safe because some patients died. There is not enough medicine in the hospital and some check-ups needed to be done outside of the hospital, this costs a lot of money.” – control participant, 23 years old, second pregnancy

“I don't know how my baby was treated. I haven't seen my baby yet at all. I don't know why, they told me I can't see him yet. I want to see him. I believe I will see him.” – MNM participant, 29 years old, third pregnancy

Fig. 1 displays additional analysis on four domains, showing the proportion of participants who commented on the matter, among those who gave a high score and a suboptimal score,

For peer review only

respectively. However, less than 10% of the participants provided any comments, except on informed choice where almost 20% of them did. Participants scoring the quality as good or very good were just as likely as participants scoring quality as suboptimal to give negative comments when asked to answer freely.

Figure 1: Good and suboptimal scores on domains of information provision, having an informed choice, having privacy and feeling to have been treated with respect, and the proportions of participants in both groups who commented on the index domains.

The following quotes further exemplify how participants who gave high quantitative scores did have critical comments on those topics:

"Someone said to me: stay where you are or I will beat you, while I was in labour. When I see that doctor now, I feel very uncomfortable." - MNM participant, 30 years old, second pregnancy > scored a 4 on 'respect'

"No privacy because even delivery was open and everyone could see" – MNM participant, 31 years old, seventh pregnancy > scored a 5 on 'privacy'

"Staff should change their attitude. There should be better rules/guidelines about how to treat patients. They should treat patients with more respect and listen to us. They should be more focused." – MNM participant, 24 years old, second pregnancy > scored a 4 on 'respect'

DISCUSSION

Main findings

For all participants, we found a delay in access to facility care to be mostly in the decision-making process. However, around one fifth of them, MNM significantly more often so, experienced barriers in reaching care, mainly of financial and logistic nature. Once in the hospital, care was given quickly. The quality of care experienced was high when scored

For peer review only

quantitatively, although one fifth had an overall suboptimal experience. Informed choice and availability of supplies were scored as suboptimal most often. MNM experienced care as suboptimal significantly more often than controls. Our expectation was that participants would more often comment negatively if they had given suboptimal scores but this did not show consistently. Generally, there was a positive quantitative assessment with critical comments expressed by a minority of the participants.

Interpretation

In our study population, MNM reported to have experienced financial barriers more often than the controls did. Previous work found that financial costs were a major obstacle in seeking non-emergency obstetric care, but not so much in emergency obstetric care,[13]. This study shows that fostering preparedness by encouraging savings or community mediated transport could shorten access to care in a significant proportion and possibly improve outcomes.

This study's methodology was mainly quantitative, but encouraged comments from the participants to enrich the numeric outcomes. Through calculations of averages we get a sense of experienced quality of care, but the valuable information for policy change really seems to come from the positive and negative extremes. The individual participants' comments reflected those extremes best, with their experiences of substandard care highlighting ample opportunities for improvement. This is most visible in their perception of quality of care which had a discrepancy between the quantitative and qualitative outcomes. We hypothesise that quantitative outcomes mainly reflect the norms in the health care system and broader society, with people adjusting their standards to what is currently available to them. Even so, qualitative data did shed a bright light on personal needs and preferences deviating from the current norm. The norm might be what it is, but that does not mean that the patient, who is depending on the health care system, finds the norm acceptable.

For peer review only

For example, the vast majority scored 'privacy' as good or very good, whilst one in every 20 participants shared how they had to share beds, there was a lack of curtains and physical examinations were executed without shielding. The quotes reproduced in the article illustrate stories on poor treatment during hospitalization that are similar to those of other women around the world,[14-17], whose expectations of smooth access, privacy, adequate provision of information, attitude and availability of supplies reflect essential elements of high-quality maternal care as standardized by the WHO,[8]. Dissatisfaction on not meeting these expectations might not be reflected directly given the propensity to normative answers in most, but can negatively impact future service utilization and, consequently, maternal health outcomes.

Our study advocates to use both quantitative and qualitative methods to get a genuine impression of patients' perspectives and experiences. Theories in the domains of social and economic welfare have argued the potential value of patients' perspectives in health research,[18], claiming how a patient's direct testimony of the experienced illness and received health care might be too positive when compared to the public's supposedly more objective opinion. The concept of adaptive preferences explains how health care, which is formally perceived as substandard, might be perceived as sufficient or good by the receiving individual at that time. We argue, though, partly in line with Nussbaum's philosophy on this, that any patient's testimony, at any time and any place, is valid in and of itself,[18,19]. Furthermore, following a human rights-based approach to maternal health care, we believe it to be quintessential, in the pursue of universal high-quality care for all, to include women and their communities in maternal health research and policy making,[20].

Strengths and limitations

This study involved a wide range and large number of patients from the maternal population in Zanzibar's main hospital, which deals with a high volume of non- and mildly complicated cases

For peer review only

as well as with, in its role of referral hospital, the most complicated cases. Nevertheless, it is not a complete representation of the patient population, leading to selection bias. We could not include women who had not reached the hospital, including those who may suffer most from financial barriers. The same applies to the exclusion of patients who were not reachable by mobile phone, which possibly excluded those suffering more from poverty and possibly having less influence in their households and in decision-making processes regarding health. Additionally, conduction of the (majority of) interviews in the hospital made the outcomes prone to information bias, discouraging people to criticize the facility they were still in and, sometimes, depending on. Furthermore, we cannot exclude moderator and reporter bias, which we tried to mitigate through a well-defined methodology and interview structure.

In the MNM group, experiences might have been more positive because of having received comprehensive care when it was needed the most, ultimately leading to them surviving life-threatening illness. Equally, experiences might have been more negative because of a more extensive exposure to the health care system and its flaws. Amongst the not so complicated cases, experiences might have been more positive because of an easier process, although these patients might have felt less surrendered to/dependent of the health care system and, therefore, freer to be critical.

Within the group of participants who commented critically beyond the set interview questions, we reached outcome saturation, thus trusting their comments to reflect a daily experienced reality and consequently providing policy makers with starting points for health care improvement.

Methodological challenges led to a percentage of the MNM during the study period to not have been interviewed and for a lower number of control participants to have been included. The final large number of included participants, however, makes us believe that this has not led to selection bias.

For peer review only

CONCLUSION

This study assessed the perception of patients on their journey to and during maternal care in Zanzibar's main health facility. Our outcomes reflect first-hand testimonies, informing us that most patients seeking care in MMH during pregnancy and childbirth seek care on time, access MMH quickly and are treated on time. A significant minority of them, though, experiences barriers, mostly financial, in reaching care, with MNM more so than controls. Quantitatively assessed, quality of care is scored high and is similar between MNM and controls. Simultaneously, a group of participants shared insightful critical perceptions. There seems to be an opportunity to distinguish between normative and personal perceptions. To continue to conduct patient-centred and setting-specific maternal health research in the pursue of improved maternal well-being, we suggest to combine quantitative and qualitative methodologies.

REFERENCES

1. Alkema L, Chou D, Hogan D, et al. Global, regional, and national levels and trends in maternal mortality between 1990 and 2015, with scenario-based projections to 2030: a systematic analysis by the UN Maternal Mortality Estimation Inter-Agency Group. *Lancet* 2016;387(10017):462-74 doi: 10.1016/S0140-6736(15)00838-7.
2. Garbysch S, Nesbitt RC, Schoeps A, et al. Does facility birth reduce maternal and perinatal mortality in Brong Ahafo, Ghana? A secondary analysis using data on 119 244 pregnancies from two cluster-randomised controlled trials. *Lancet Glob Health* 2019;7:e1074–87.
3. World Health Organization. Quality, equity, dignity: the network to improve quality of care for maternal, newborn and child health – strategic objectives. Geneva: WHO 2018.
4. Bohren MA, Mehrtash H, Fawole B, et al. How women are treated during facility-based childbirth in four countries: a cross-sectional study with labour observations and community-based surveys. *Lancet* 2019;394:1750–1763.

For peer review only

5. Solnes Miltenburg A, van Pelt S, Meguid T,et al. Disrespect and abuse in maternity care: individual consequences of structural violence. *Reprod Health Matters* 2018;26(53):88-106 doi:10.1080/09688080.2018.1502023.
6. Ministry of Health, Revolutionary Government of Zanzibar. Zanzibar Annual Health Bulletin. Zanzibar, United Republic of Tanzania 2016.
7. Herklots T, van Acht L, Khamis RS, et al. Validity of WHO's near-miss approach in a high maternal mortality setting. *PLoS ONE* 2019;14(5):e0217135 doi: 10.1371/journal.pone.0217135.
8. World Health Organization. Standards for improving quality of maternal and newborn care in health facilities. Geneva: WHO 2016.
9. Fakih B, Azzah Nofly AS, Ali AO, et al. The status of maternal and newborn health care services in Zanzibar. *BMC Pregnancy and Childbirth*. 2016;16:134 doi: 10.1186/s12884-016-0928-6.
10. Gon G, Ali SM, Towriss C, et al. Unpacking the enabling factors for hand, cord and birth-surface hygiene in Zanzibar maternity units. *Health Policy Plan* 2017;32:1220-1228 doi: 10.1093/heapol/czx081.
11. World Medical Association Declaration of Helsinki: ethical principles for medical research involving human subjects. *JAMA* 2013;310(20):2191-2194 doi:10.1001/jama.2013.281053.
12. Herklots T, van Acht L, Meguid T, et al. Severe maternal morbidity in Zanzibar's referral hospital: Measuring the impact of in-hospital care. *PLoS One* 2017;12(8):e0181470 doi: 10.1371/journal.pone.0181470.
13. Norhayati MN, Surianti S, Hussain Nik Hazlina N. Metasynthesis: Experiences of women with severe maternal morbidity and their perception of the quality of health care. *PLoS One* 2015;10(7): e0130452 doi: 10.1371/journal.pone.0130452.
14. Tunçalp Ö, Hindin MJ, Adu-Bonsaffoh K, et al. Listening to women's voices: The quality of care of women experiencing severe maternal morbidity, in Accra, Ghana. *PLoS One* 2012;7(8): e44536 doi: 10.1371/journal.pone.0044536.

For peer review only

15. Rööst M, Jonsson C, Liljestrand J, et al. Social differentiation and embodied dispositions: a qualitative study of maternal care-seeking behaviour for near-miss morbidity in Bolivia. *Reprod Health* 2009;29;6:13 doi: 10.1186/1742-4755-6-13.

16. Weeks A, Lavender T, Nazziwa E, et al. Personal accounts of 'near-miss' maternal mortalities in Kampala, Uganda. *BJOG* 2005;112(9):1302-7.

17. Norhayati MN, Nik Hazlina NH, Ab Razak A, et al. The experiences of women with maternal near miss and their perception of quality of care in Kelantan, Malaysia: a qualitative study. *BMC Pregnancy and Childbirth* 2017;17:189 doi: 10.1186/s12884-017-1377-6.

18. Nussbaum M. Symposium on Amartya Sen's philosophy: Adaptive preferences and women's options. *Economics & Philosophy* 2001; 17(1):67-88 doi: 10.1017/S0266267101000153.

19. Mitchell P. Adaptive Preferences, Adapted Preferences. *Mind* 2018; 127(508):1003-1025 doi: 10.1093/mind/fzy020.

20. Freedman LP. Using human rights in maternal mortality: from analysis to strategy. *Int J Gynaecol Obstet* 2001;75:51-60.

FIGURE LEGEND

Figure 1. Legend: top bar left side= suboptimal score with no comment; top bar right side= suboptimal score with comment; bottom bar left side= good score with no comment; bottom bar right side= good score with comment

SUPPLEMENTARY FILES

Table 1: Markers for organ dysfunction, identifying cases in life-threatening condition

Table 2: Interview outline

For peer review only

AUTHOR CONTRIBUTIONSHIP

TH, TM and BJ conceptualized the study. TH, KMS, MR and LD conducted the majority of the data collection and were responsible for data integrity. TH, SC and MPHK performed data analysis. TH and TM supervised the research on-site. BJ and AF supervised the research off-site. TH drafted the manuscript. All authors revised multiple versions of the manuscript. All authors have read and approved the final version of the manuscript.

COMPETING INTERESTS

None declared.

ETHICAL APPROVAL

Ethical approval was obtained from Zanzibar’s Medical Research and Ethics Committee under protocol reference number ZAMREC/0002/JUN/17.

DATA SHARING STATEMENT

No additional data available

FUNDING

No specific funding was received for this study

PATIENT AND PUBLIC INVOLVEMENT

For peer review only

The lack of patient's perspectives in maternal health research in our setting motivated our research questions and methodology. Patients and public were not directly involved in the design of the study, nor in the plans for dissemination. The flexible construct of the interviews did allow the patient to decide on which topics she wanted to elaborate. Dissemination of the study outcomes will, next to publication in an open access scientific journal, take place in the study setting, to the index hospital and to Zanzibar's Ministry of Health. We hope this study can inspire the local research agenda, motivating patient involvement.

ACKNOWLEDGEMENTS

We thank Zuwena Ali Rashid, Vreni Bron, Suhaila Salum Yussuf, Sigrid Hilgen, Marloes Plender, Lisa Trommelen, Jenna Uijt de Haag, Jasmijn Rake, Jasmijn Broerze, Ibrahim Shaban, Elleke van der Meij, Eline Veenstra and Claire Bausch for their enthusiastic, tireless contributions to the data collection. We thank all health workers of Mnazi Mmoja Hospital's departments of Obstetrics & Gynaecology, Internal Medicine, Surgery and Intensive Care for facilitating and supporting participant inclusion and data collection. We thank the administration of Mnazi Mmoja Hospital for facilitating and supporting this study. We are deeply grateful to all the participants for opening up and sharing with us their opinions and ideas.

For peer review only

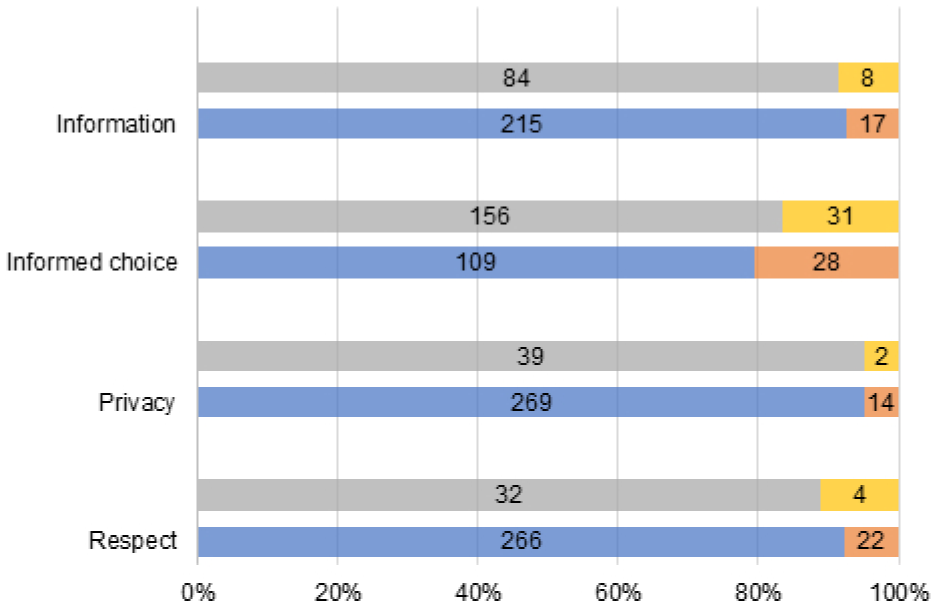

87x59mm (144 x 144 DPI)

Table 1: Markers for organ dysfunction, identifying cases in life-threatening condition

Type of organ dysfunction	Markers (clinical signs, laboratory markers and management actions)
**Cardiovascular**	Shock[1], use of continuous vasoactive drugs, cardiac arrest, cardio-pulmonary resuscitation, severe hypoperfusion (lactate >5 mmol/L or >45mg/dL)[2] or severe acidosis (pH <7.1)[2]
**Respiratory**	Acute cyanosis, gasping[3], severe tachypnea (respiratory rate >40bpm), severe bradypnea (respiratory rate <6bpm), severe hypoxemia (PAO2/FiO2 <200mmHg[2] or O2 saturation <90% for ≥60min) or intubation and ventilation not related to anaesthesia
**Renal**	Oliguria[4] non-responsive to fluids or diuretics, dialysis for acute renal failure[5] or severe acute azotaemia (creatinine ≥300umol/ml or ≥3.5mg/dL)
**Coagulation / haematological**	Clotting failure[6], use of continuous vasoactive drugs[7], massive transfusion of blood or red cells (≥5 units)[8] or severe acute thrombocytopenia (<50,000 platelets/ml)
**Hepatic**	Jaundice in the presence of pre-eclampsia[9], severe acute hyperbilirubinemia (bilirubin >100umol/L or >6.0mg/dL)
**Neurological**	Prolonged unconsciousness (lasting >12 hours)/coma[10], stroke[11], status epilepticus[12], uncontrollable fits/total paralysis
**Uterine**	Hysterectomy following haemorrhage or infection

1) Shock is a persistent severe hypotension, defined as a systolic blood pressure <90 mmHg for ≥60 minutes with a pulse rate at least 120 despite aggressive fluid replacement (>2l)
2) Laboratory test or management intervention that is not available at MMH
3) Gasping is a terminal respiratory pattern and the breath is convulsively and audibly caught
4) Oliguria is defined as a urinary output <30 ml/hr for 4 hours or <400 ml/24 hr
5) Dialysis services have been available in MMH since 26 May 2017
6) Clotting failure can be assessed by the bedside clotting test or absence of clotting from the intravenous site after 7–10 minutes
7) For instance, continuous use of any dose of dopamine, epinephrine or norepinephrine
8) In MMH extended to include all types of blood products and cases in which 5 or more units were requested but not given due to shortage
9) Pre-eclampsia is defined as the presence of hypertension associated with proteinuria. Hypertension is defined as a blood pressure of at least 140 mmHg (systolic) or at least 90 mmHg (diastolic) on at least two occasions and at least 4–6 h apart after the 20th week of gestation in women known to be normotensive beforehand. In MMH, proteinuria is defined as ≥2+ protein on dipstick.
10) Loss of consciousness is a profound alteration of mental state that involves complete or near-complete lack of responsiveness to external stimuli. It is defined as a Coma Glasgow Scale <10 (moderate or severe coma).
11) Stroke is a neurological deficit of cerebrovascular cause that persists beyond 24 hours or is interrupted by death within 24 hours
12) Condition in which the brain is in a state of continuous seizure

For peer review only

Table 2: Interview outline

<table>
<tr><td colspan="9">PATIENT INTERVIEW – BEFORE DISCHARGE (English version)</td><td colspan="2">NO.:</td></tr>
<tr><td>Topic</td><td colspan="10">Questions</td></tr>
<tr><td rowspan="5">1) Patient characteristics</td><td>Age:</td><td colspan="9">Level of education (primary, secondary, tertiary):</td></tr>
<tr><td>Gravidity/Parity:</td><td colspan="9">Marital status (first-, second-, third- or fourth wife, divorced, widow, single):</td></tr>
<tr><td>Number of living children:</td><td colspan="9">Perceived wealth:
Very poor – Poor – Average – Rich – Very Rich</td></tr>
<tr><td>Number of children of school-going age attending school:</td><td colspan="9">Occupation:
Paid work – Self-employed – Non-paid work – Student – Housework – Unemployed for health reason – Unemployed for other reason – Other, …………………</td></tr>
<tr><td>Smoking?</td><td>Alcohol?</td><td colspan="8">Drugs?</td></tr>
<tr><td>2) Family Planning</td><td>Pregnancy planned?</td><td>If unplanned, wanted?</td><td colspan="8">Did you have access to contraceptives?</td></tr>
<tr><td colspan="3"></td><td></td><td>1</td><td>2</td><td>3</td><td>4</td><td>5</td><td>na</td></tr>
<tr><td rowspan="7">3) Antenatal Care (ANC)</td><td colspan="3">How did you experience the access of ANC? Scale: 1=impossible, 2=difficult, 3=not simple, not difficult, 4=simple, 5= very simple, na=not applicable</td><td></td><td></td><td></td><td></td><td></td><td></td></tr>
<tr><td colspan="3">How do you feel about your overall experience in ANC? Scale: 1=very dissatisfied, 2=dissatisfied, 3=neither satisfied, nor dissatisfied, 4=satisfied, 5= very satisfied, na=not applicable</td><td></td><td></td><td></td><td></td><td></td><td></td></tr>
<tr><td colspan="9">Did you consult others for treatment during your pregnancy such as traditional birth attendants or others?</td></tr>
<tr><td colspan="2">a. No</td><td colspan="7"></td></tr>
<tr><td colspan="2">b. Yes, traditional birth attendant</td><td colspan="7"></td></tr>
<tr><td colspan="2">c. Yes, other</td><td colspan="7"></td></tr>
<tr><td colspan="9">Comments:</td></tr>
<tr><td rowspan="16">4) Access to Health Facility</td><td colspan="9">Where did you plan to deliver?</td></tr>
<tr><td colspan="2">a. Home</td><td colspan="7"></td></tr>
<tr><td colspan="2">b. Health center</td><td colspan="7"></td></tr>
<tr><td colspan="2">c. Other hospital</td><td colspan="7"></td></tr>
<tr><td colspan="2">d. MMH</td><td colspan="7"></td></tr>
<tr><td colspan="9">What was the <u>main</u> motivation for delivering there?</td></tr>
<tr><td colspan="9">Who accompanied you to the health facility and took care of you during hospitalization?</td></tr>
<tr><td colspan="2">a. Female family member</td><td colspan="7"></td></tr>
<tr><td colspan="2">b. Husband</td><td colspan="7"></td></tr>
<tr><td colspan="2">c. Friend</td><td colspan="7"></td></tr>
<tr><td colspan="2">d. Other</td><td colspan="7"></td></tr>
<tr><td colspan="9">Who took care of your children during your hospitalization?</td></tr>
<tr><td colspan="2">a. Female family member</td><td colspan="7"></td></tr>
<tr><td colspan="2">b. Husband</td><td colspan="7"></td></tr>
<tr><td colspan="2">c. Friend</td><td colspan="7"></td></tr>
<tr><td colspan="2">d. Other</td><td colspan="7"></td></tr>
</table>

For peer review only

		1	2	3	4	5	na
	**How did you experience the access to the health facility?** Scale: 1=very poor, 2=poor, 3=not good, not poor, 4=good, 5= very good, na=not applicable						
	**If you encountered problems, what kind of problems?**						
	a. Financial						
	b. Referral						
	c. Logistic						
	d. No problems						
	**How did you come here and how long did that take?**						
	**Comments:**						
**5) Perception on Development of Disease/ Complications**	**How much time passed between when you started feeling less well and you sought medical help?**						
	**How much time passed between when you arrived in the hospital/you started feeling less well in the hospital and the moment a health worker came to see/question/examine you?**						
	**Did you recognise that you started to feel worse? If so, how did you feel and what did you think?**						
		1	2	3	4	5	na
	**Did you have knowledge about your disease/ complication(s) before you suffered from it yourself? If so, how?** Scale: 1=not at all, 2= little, 3= moderate amount, 4=good knowledge, 5=extreme good knowledge, na=not applicable						
	**Comments**						
		1	2	3	4	5	na
**6) Perceived Quality of Care during Hospitalization**	**Did you feel treated with respect?** Scale: 1=not at all, 2=a little, 3=a moderate amount, 4=very much, 5=extremely, na=not applicable						
	**Did you feel informed about treatments/ complications?** Scale: 1=not at all, 2=a little, 3=a moderate amount, 4=very much, 5=extremely, na=not applicable						
	**Did you feel you had an informed choice in the services you received?** Scale: 1=not at all, 2=a little, 3=a moderate amount, 4=very much, 5=extremely, na=not applicable						
	**Did you feel you had privacy?** Scale: 1=not at all, 2=a little, 3=a moderate amount, 4=very much, 5=extremely, na=not applicable						
	**Were you able to receive emotional support from relatives/friends?** Scale: 1=not at all, 2=a little, 3=a moderate amount, 4=very much, 5=extremely, na=not applicable						
	**Did you feel you were provided with emotional support by the staff?** Scale: 1=not at all, 2=a little, 3=a moderate amount, 4=very much, 5=extremely, na=not applicable						

For peer review only

		1	2	3	4	5	na
	**Did you feel that the staff was motivated and available?** Scale: 1=not at all, 2=a little, 3=a moderate amount, 4=very much, 5=extremely, na=not applicable						
	**Did you feel safe in the hospital?** Scale: 1=not at all, 2=a little, 3=a moderate amount, 4=very much, 5=extremely, na=not applicable						
	**Did you feel the health workers took time for you?** Scale: 1=not at all, 2=a little, 3=a moderate amount, 4=very much, 5=extremely, na=not applicable						
	**Did you experience financial barriers to getting the right treatment?** Scale: 1=extremely, 2=very much, 3=a moderate amount, 4=a little, 5=not at all, na=not applicable						
	**Did you think the hospital had sufficient supplies to care for you? (Sanitation, medicine, equipment)** Scale: 1=not at all, 2=a little, 3=a moderate amount, 4=very much, 5=extremely, na=not applicable						
	**How do you feel about the way your baby was treated?** Scale: 1=very dissatisfied, 2=dissatisfied, 3=neither satisfied, nor dissatisfied, 4=satisfied, 5= very satisfied, na=not applicable						
	**How do you feel about the overall experience of your admission?** Scale: 1=very dissatisfied, 2=dissatisfied, 3=neither satisfied, nor dissatisfied, 4=satisfied, 5= very satisfied, na=not applicable						
	If your baby died:						
		**1**	**2**	**3**	**4**	**5**	**na**
	**How do you feel about the way you were informed on the death of your baby?** Scale: 1=very dissatisfied, 2=dissatisfied, 3=neither satisfied, nor dissatisfied, 4=satisfied, 5= very satisfied, na=not applicable						
	**How do you feel about the way you, your baby and the baby's father were treated after your baby's death?** Scale: 1=very dissatisfied, 2=dissatisfied, 3=neither satisfied, nor dissatisfied, 4=satisfied, 5= very satisfied, na=not applicable						
	**Comments:**						
	**SCALE** 1=an extreme impact, 2= a high impact, 3=a moderate impact, 4= little impact, 5= no impact at all, na=not applicable	**1**	**2**	**3**	**4**	**5**	**na**
**7) Perspective on Impact of Hospitalization**	**Impact on your position within your close family**						
	**Impact on your position within extended family/community**						
	**Financial and economic impact of hospitalization**						
	**Impact on educational prospects your children**						
	**Comments:**						
**8) State upon Discharge**	**Do you feel recovered at discharge?** Yes/No						
	**Do you feel able to resume your daily activities?** Yes/No						

For peer review only

**9) Recommend**	**Would you recommend your sister to deliver in the same place?** Yes/No
**10) Suggestions**	**Do you have any suggestions to improve the care for you and your baby in the future? What would you like to see different?**

For peer review only

BMJ Open

The journal to facility birth in Zanzibar: a questionnaire-based cohort study of patients' perspectives on preparedness, access and quality of care

Journal:	*BMJ Open*
Manuscript ID	bmjopen-2020-040381.R1
Article Type:	Original research
Date Submitted by the Author:	31-Aug-2020
Complete List of Authors:	Herklots, Tanneke; Erasmus MC, Obstetrics & Gynaecology<br>D'haene, Lara; Hospital Doctor Jose Molina Orosa, Emergency Medicine<br>Mbarouk, Khairat; Mnazi Mmoja Referral Hospital, Obstetrics & Gynaecology<br>Rajhy, Mubina; Mnazi Mmoja Referral Hospital, Obstetrics & Gynaecology<br>Couperus, Simone; University of Utrecht Faculty of Medicine, Obstetrics &Gynaecology<br>Meguid, Tarek ; Village Health Works<br>Franx, Arie; Erasmus MC, Obstetrics & Gynaecology<br>Koster, Maria PH; Erasmus MC, Obstetrics & Gynaecology<br>Jacod, Benoit; Onze Lieve Vrouwe Gasthuis, Obstetrics & Gynaecology
**Primary Subject Heading**:	Obstetrics and gynaecology
Secondary Subject Heading:	Global health, Patient-centred medicine, Reproductive medicine
Keywords:	Maternal medicine < OBSTETRICS, Quality in health care < HEALTH SERVICES ADMINISTRATION & MANAGEMENT, Organisation of health services < HEALTH SERVICES ADMINISTRATION & MANAGEMENT, REPRODUCTIVE MEDICINE

SCHOLARONE™
Manuscripts

I, the Submitting Author has the right to grant and does grant on behalf of all authors of the Work (as defined in the below author licence), an exclusive licence and/or a non-exclusive licence for contributions from authors who are: i) UK Crown employees; ii) where BMJ has agreed a CC-BY licence shall apply, and/or iii) in accordance with the terms applicable for US Federal Government officers or employees acting as part of their official duties; on a worldwide, perpetual, irrevocable, royalty-free basis to BMJ Publishing Group Ltd ("BMJ") its licensees and where the relevant Journal is co-owned by BMJ to the co-owners of the Journal, to publish the Work in this journal and any other BMJ products and to exploit all rights, as set out in our licence.

The Submitting Author accepts and understands that any supply made under these terms is made by BMJ to the Submitting Author unless you are acting as an employee on behalf of your employer or a postgraduate student of an affiliated institution which is paying any applicable article publishing charge ("APC") for Open Access articles. Where the Submitting Author wishes to make the Work available on an Open Access basis (and intends to pay the relevant APC), the terms of reuse of such Open Access shall be governed by a Creative Commons licence – details of these licences and which Creative Commons licence will apply to this Work are set out in our licence referred to above.

Other than as permitted in any relevant BMJ Author's Self Archiving Policies, I confirm this Work has not been accepted for publication elsewhere, is not being considered for publication elsewhere and does not duplicate material already published. I confirm all authors consent to publication of this Work and authorise the granting of this licence.

For peer review only

The journey to facility birth in Zanzibar: a questionnaire-based cohort study of patients' perspectives on preparedness, access and quality of care

Tanneke Herklots[1*], Lara D'haene[2], Khairat Mbarouk Said[3], Mubina Rajhy[3], Simone Couperus[4], Tarek Meguid[5], Arie Franx[1], Maria PH Koster[1], Benoit Jacod [6]

1. Department of Obstetrics & Gynaecology, Erasmus MC, University Medical Centre Rotterdam, Rotterdam, The Netherlands
2. Department of Emergency Medicine, Hospital Doctor Jose Molina Orosa, Lanzarote, Spain
3. Department of Obstetrics & Gynaecology, Mnazi Mmoja Hospital, Zanzibar, United Republic of Tanzania
4. Department of Obstetrics & Gynaecology, University Medical Centre Utrecht, Utrecht, The Netherlands
5. Village Health Works, Kigutu, Burundi
6. Department of Obstetrics & Gynaecology, Onze Lieve Vrouw Gasthuis, Amsterdam, The Netherlands

*) Corresponding author: full name: Tanneke Herklots; postal address: Kromme Slagen 11, 4823 LL, Breda, The Netherlands; e-mail: tannekeherklots@gmail.com

Word count abstract: 300

Word count full text: 3507

For peer review only

ABSTRACT

Introduction

Tackling substandard maternity care in health facilities requires engaging women's perspectives in strategies to improve outcomes. This study aims to provide insights in the perspectives of women with severe maternal morbidity on preparedness, access and quality of care in Zanzibar's referral hospital.

Methods

In a prospective cohort from April 2017 until December 2018, we performed semi-structured interviews with women who experienced maternal near-miss complications and matched controls. These focused on sociodemographic and obstetric characteristics, perceived accessibility to and quality of facility care with 15 domains, scored on a one-to-five scale. Participants' comments and answers to open questions were employed to illustrate quantitative outcomes. Zanzibar's Medical Research and Ethics Committee approved the study (ZAMREC/0002/JUN/17).

Results

We included 174 cases and 151 controls. Compared to controls, patients with a near-miss had less formal education (p=0.049), perceived their wealth as poor (p=0.002), and more often had a stillbirth (p<0.001). Many experienced a delay in deciding to seek care. More than controls, near-miss patients experienced barriers in reaching care (p=0.049), often of financial nature (13.8% vs. 4.0%). Quality of care was perceived as high, with means above three out of five, in 14 out of 15 domains. One fifth had an overall suboptimal experience, mostly regarding informed choice and supplies availability. Additional comments were expressed by a minority of participants.

Conclusion

Most patients promptly sought, accessed and received maternity care in Zanzibar's referral hospital. A minority experienced barriers, mostly financial, in reaching care, and more so among patients with near-miss complications. Quality of facility care was generally highly

For peer review only

rated. However, a group did report insightful critical perceptions. This study highlights the necessity to mitigate effects of sociodemographic differences on health, to involve patients in decisions regarding maternity care, and to ensure availability of medical supplies, all which will contribute to improved maternal well-being.

STRENGTHS AND LIMITATIONS OF THIS STUDY

- The study involved a wide range and large number of maternal patients in Zanzibar's main hospital
- Because we could not include women who had not reached the hospital, nor patients who were not in possession of a mobile phone, the study had a selection bias potentially at the cost of those suffering more from poverty
- There might have been a certain level of information bias because the (majority of) interviews was conducted in the hospital, potentially discouraging people to criticize the facility they were at
- The given comments reached outcome saturation, for which we trust them to experienced reality, thus being valid starting points for healthcare improvement to provide to policy makers
- This study displays how facility maternity care is perceived by the patients themselves, and does so in an extensive, profound and both quantitative and qualitative manner

For peer review only

INTRODUCTION

Low-income countries carry a disproportionately large burden of maternal morbidity and mortality. Efforts have gone into ensuring availability of essential interventions, promoting facility deliveries and skilled birth attendance,[1]. Unfortunately, despite a widespread increase in facility deliveries, the improvement in pregnancy outcomes has been slower than expected. This might be because women and their families are not timely prepared to seek facility care or face limitations in accessing facility care, but there seems to be a causal role for the low quality of care provided in many facilities,[2]. Low quality of care negatively affects the willingness to seek facility care, either in a future pregnancy or of other pregnant women in the community. Poor quality of care is not limited to but includes maltreatment during childbirth, which has been found to be common in many low-income settings,[3-5]. Tackling substandard maternity care requires incorporating the perspectives of women in designing strategies to improve maternal and child outcomes.

A specific group of women within the health care system are those who experience life-threatening complications during pregnancy and childbirth, so-called maternal near-miss complications, but survive. Maternal deaths are an often-used proxy for assessing the quality of care but in the group of all child-bearing women, represent an absolute small number. Women who had an MNM represent a larger proportion, whilst still being a group small enough to accommodate in-depth assessments of their multi-facetted experiences in the health care system.

In Zanzibar, Tanzania, around half of the deliveries take place at home and half in a health facility,[6]. Of the latter, around 30% take place in the region's main public referral health facility: Mnazi Mmoja Hospital (MMH). The maternal mortality rate (MMR) at MMH is around 400 per 100,000 livebirths,[7]. Multiple quality of care assessments have been performed at Tanzanian health care facilities guided by the World Health Organization's (WHO) Standard for Care,[8]. In 2012, a large unmet need for basic and comprehensive emergency obstetric and neonatal care was found throughout Zanzibar, including shortages of staff and supplies,

and an insufficient geographical access,[9]. In 2016, an assessment in Zanzibar showed that five main governmental hospitals, including MMH, were overall not conducive for high-quality maternal and child health care provision (Meguid T, Mrisho Haji F, Herklots T, Rajhy M, Kassim NI. Conduciveness for High Quality of Maternal- and Child Health Care in Hospitals in Zanzibar. 2016). In 2017, an assessment of 37 maternity units in Zanzibar showed how more than half of those had challenges in providing hand and surface hygiene,[10].

This objective of this study is to explore the experiences of women with and without near-miss complications regarding their preparedness for, access to and quality of facility care in Zanzibar's main hospital. Understanding maternity care with its qualities and flaws from the patients' perspectives will allow to effectively inform local and regional maternal health policies and research agendas.

METHODS

From April 2017 until December 2018, we conducted a prospective cohort study at the department of Obstetrics & Gynaecology of MMH. We performed semi-structured interviews with women who experienced a maternal near-miss (MNM) and control participants. MNM were identified during the department's twice daily meetings by a locally adapted and validated version of the WHO near-miss approach (for criteria see Supplementary Files, Table 1),[7]. Controls, matched on a 1:1 ratio, did not have a severely complicated pregnancy and had a similar date of admission, similar mode of delivery and similar gestational age as MNM.

This research was approved by Zanzibar's Medical Research and Ethics Committee (ZAMREC/0002/JUN/17) and conducted in line with the principles of the Declaration of Helsinki,[11]. In addition to providing informed consent, inclusion criteria were age of 18 years or above, no severe pre-diagnosed psychiatric disorders, residence on Unguja (Zanzibar's main island on which MMH is located) and to be reachable by mobile phone. Informed consent of all participants was obtained in writing or verbally, in case of illiteracy. An interview was performed before discharge or, in case of logistic restrictions, within one week of discharge at

For peer review only

the participant's home. The interviews were performed by a researcher and a mediator/translator, both not involved in the clinical care of the woman. The interview comprised the following: sociodemographic circumstances (age, self-reported level of formal education, type of occupation, marital status, perceived wealth), obstetric characteristics (parity – with a grand multiparity defined as a parity above 4, singleton or multiple pregnancy, gestational age, planned location for delivery and pregnancy outcome being abortion, live birth or still birth), utilization and opinion of antenatal care (ANC) services (not included in this publication), perception on the accessibility of facility care and perception on the quality of facility care (see Supplementary Files, Table 2, for the interview outline). Considering the latter two metrics, the interviewer proactively requested the interviewees' comments in addition to the quantitative scores. This yielded quotes which we combined with the quantitative outcomes.

SPSS Statistics (version 25) and OpenEpi (version 3.01) were used for statistical analysis. Numeric outcomes were categorized similarly as in previous studies in this setting: age younger than 20 years, 20 to 35 years and older than 35 years; and parity zero, one to four or higher than four,[7,12]. Baseline characteristics were compared using Pearson's chi-square tests and, in case of small sample sizes, Fisher's exact test. Regarding quality of care, the interview outcomes were on a five-point scale, from which means per question were calculated and compared between MNM and controls using Mann-Whitney-U-tests. Suboptimal care was defined as a score of 3 or lower. Proportions of participants grading the assessed element as suboptimal were reflected in percentages. Odds ratios (OR) with 95% confidence intervals (CI) were calculated to compare between MNM and controls, to assess if having had a pregnancy with a life-threatening complication affects quality of care perception. We observed a discrepancy between the fact that the majority of patients perceived quality of care as good or very good and the fact that give comments most often had a negative connotation. To investigate this discrepancy further, we compared the frequency of negative comments in participants having scored as positive or as suboptimal on the domain to which the comments

For peer review only

pertain. We deemed the number of participants giving comments to be sufficiently high to perform this analysis on four domains: information, informed choice, respect and privacy.

A p-value below 0.05 was considered statistically significant. Quotations taken from participants' comments and answers to open questions were employed to illustrate the quantitative outcomes.

Patient and public involvement

The lack of patient's perspectives in maternal health research in our setting motivated our research questions and methodology. Patients and public were not directly involved in the design of the study, nor in the plans for dissemination. The flexible construct of the interviews did allow the patient to decide on which topics she wanted to elaborate. Dissemination of the study outcomes will, next to publication in an open access scientific journal, take place in the study setting, to the index hospital and to Zanzibar's Ministry of Health. We hope this study can inspire the local research agenda, motivating patient involvement.

RESULTS

During the study period, 269 patients were identified as MNM, of whom 174 (64.7%) were interviewed. Reasons for no study participation were patients' personal reasons, meeting one of the exclusion criteria or an unexpected early discharge home. A total of 151 control participants were interviewed.

Participants' characteristics

Sociodemographic and obstetric baseline characteristics are displayed in Table 1. MNM and controls were comparable at baseline regarding age, occupation and marital status. MNM were significantly more likely to have received primary education only (29.9% vs. 19.2%, p=0.05), more often perceived their own wealth as very poor or poor (19.0% vs. 7.3%, p=0.002), were less often nulliparous (32.2% vs. 50.3%, p=0.002), and more often grand multiparous (13.9%

For peer review only

vs. 6.0%, p=0.002). Of the MNM pregnancies, 42.9% ended in a stillbirth compared to 5.4% of control pregnancies (p<0.001). Nearly two-thirds of all participants had initially planned to deliver at MMH, controls significantly more so than MNM (total 66.5%; 58.6% MNM vs. 75.5% controls, p=0.004).

Table 1: Sociodemographic and obstetric baseline characteristics

	**Total N (%)**	**MNM N (%)**	**Controls N (%)**	**p-value**
**Total**	325 (100)	174 (53.5)	151 (46.5)	
**Age**				0.25
Data available[1]	324 (99.7)	174 (100)	150 (99.3)	
<20 years	16 (4.9)	11 (6.3)	5 (3.3)	
20-35 years	267 (82.4)	138 (79.3)	129 (86.0)	
>35 years	41 (12.7)	25 (14.4)	16 (10.7)	
**Parity**				**0.002**
Data available[1]	324 (99.7)	173 (99.4)	151 (100)	
0 (nulliparous)	132 (40.7)	56 (32.4)	76 (50.3)	
1-4 (multiparous)	159 (49.1)	93 (53.8)	66 (43.7)	
5 or more (grand multiparous)	33 (10.2)	24 (13.9)	9 (6.0)	
**Formal education level**				**0.049**
No formal education	24 (7.4)	15 (8.6)	9 (6.0)	
Primary	81 (24.0)	52 (29.9)	29 (19.2)	
Secondary	194 (59.7)	97 (55.7)	97 (64.2)	
Higher	26 (8.0)	10 (5.7)	16 (10.6)	
**Occupation**				0.80
Paid work or self-employed	148 (45.5)	76 (43.7)	72 (47.7)	
Housework	163 (50.2)	91 (52.3)	72 (47.7)	
Non-paid work or student	9 (2.8)	5 (2.9)	4 (2.6)	
Unemployed	5 (1.5)	2 (1.1)	3 (2.0)	
**Marital status**				0.21
Married	299 (92.0)	157 (90.2)	142 (94.0)	
Unmarried	26 (8.0)	17 (9.8)	9 (6.0)	
**Perceived wealth**				**0.002**
Very poor or poor	44 (13.5)	33 (19.0)	11 (7.3)	
Average	280 (86.2)	140 (80.5)	140 (92.7)	
Rich or very rich	1 (0.3)	1 (0.6)	0 (0.0)	
**Singleton/multiple pregnancy[2]**				**0.03**
Data available	261 (80.3)	144 (82.8)	117 (77.5)	
Singleton	242 (92.7)	129 (89.6)	113 (96.6)	
Multiple	19 (7.3)	15 (10.4)	4 (3.4)	

For peer review only

Planned location for delivery				0.004
MMH	216 (66.5)	102 (58.6)	114 (75.5)	
Other hospital	97 (29.8)	63 (36.2)	34 (22.5)	
Health centre	8 (2.5)	6 (3.4)	2 (1.3)	
Home	4 (1.2)	3 (1.7)	1 (0.7)	
**Pregnancy outcome**				**<0.001**
Data available[1]	319 (98.2)	170 (97.7)	149 (98.7)	
Ending before 20 weeks	43 (13.5)	25 (14.7)	18 (12.1)	
Live birth	191 (59.9)	69 (40.6)	122 (81.9)	
Stillbirth	81 (25.4)	73 (42.9)	8 (5.4)	
Still pregnant at discharge	4 (1.3)	3 (1.8)	1 (0.7)	

1) In case of missing data, the table shows the available data per category in the first row; the consequent proportions are calculated with the available data as denominator
2) This excludes participants who were admitted with a gestational age below 20 weeks, including pregnancies which ended in abortion and ectopic pregnancies

Access to care

Motivations for choosing a delivery location did not differ between MNM and controls and were most often because of previous experience and expectations (36.6%) or medical indication (28.0%).

"I didn't decide yet in which hospital but I don't want to deliver at home because you can have a problem and not be able to solve it" – MNM participant, 25 years old, first pregnancy

The majority of MNM (59.2%) initiated their journey to MMH because they recognised that their condition was deteriorating, whilst less than a quarter (23.0%) of them said they were unaware.

"I recognized it was not good. I thought I needed to have an operation because that is what happened to my sister-in-law." – control participant, 36 years old, fourth pregnancy

Table 2 displays the various elements on access to care investigated. Over half of the MNM (55.6%) and the controls (66.1%) said to have taken more than two hours before they decided to seek medical care after starting to feel sick or labour started (p=0.18). After that, it took 36.2% of MNM and 29.1% of controls longer than 30 minutes to reach a health facility (p=0.10). Once arrived, the vast majority was attended to promptly, but some had waited longer than three hours, and MNM significantly more often than controls (9.3% vs. 5.4%, p=0.04).

Close to one fifth of all interviewees (18.6%) experienced the access to care as suboptimal, with a significant higher number of MNM than controls reporting to have encountered problems of financial, logistical or referral-related nature (22.4% MNM vs 13.9% controls, p=0.049). Many interviewees shared that they had struggled with finding and paying petrol, a borrowed car or public transportation.

"I had to wait and find a bus for six hours. There should be transport available" – MNM participant, 19 years old, second pregnancy

"I came by ambulance from [my area] and paid 10,000TSH (equivalent to 5 USD, authors). I find it not fair of them that I had to pay. What if I had no money?" – MNM participant (area of residence to MMH around a 20-minutes-drive, 8-9km), 40 years old, sixth pregnancy

"Because my husband was not able to buy petrol for our car, I took a dala dala (public mini bus, authors) for three hours from [my area] to MMH." – MNM participant (area of residence to MMH around one and a half hour-drive by car, 40km), 38 years old, eight pregnancy

Table 2: Experience of accessibility of care

	Total N (%)	MNM N (%)	Controls N (%)	p-value[1]
**Total**	325 (100.0)	174 (53.5)	151 (46.5)	
**Sought care X time after complaints started *(delay 1)***				0.18
Data available[2]	284 (87.4)	151 (86.8)	133 (88.1)	

For peer review only

Immediately	60 (21.1)	40 (26.5)	20 (15.0)	
<2 hours	35 (12.3)	19 (12.6)	16 (12.0)	
2-8 hours	96 (33.8)	45 (29.8)	51 (38.3)	
>8 hours	76 (26.8)	39 (25.8)	37 (27.8)	
Not applicable	17 (6.0)	8 (5.3)	9 (6.8)	
**Reached care X time after start of journey *(delay 2)***				0.10
Data available[2]	261 (80.3)	129 (74.1)	132 (87.4)	
<15min	70 (25.7)	36 (26.1)	34 (25.4)	
15-30min	102 (37.5)	43 (31.2)	59 (44.0)	
30-60min	49 (18.0)	24 (17.4)	25 (18.7)	
>60min	40 (14.7)	26 (18.8)	14 (10.4)	
**Received care X time after arrival in health facility *(delay 3)***				**0.04**
Data available[2]	304	158	146	
Immediately	251 (78.7)	122 (71.3)	129 (87.2)	
<3hrs	29 (9.1)	20 (11.7)	9 (6.1)	
>3hrs	24 (7.5)	16 (9.3)	8 (5.4)	
**Experienced access to care**				0.48[3]
Data available[2]	322	171	151	
Very poor	2 (0.6)	2 (1.2)	-	
Poor	10 (3.1)	5 (2.9)	5 (3.3)	
Not poor, not good	48 (14.9)	28 (16.4)	20 (13.2)	
Good	59 (18.3)	35 (20.5)	24 (15.9)	
Very good	203 (63.0)	101 (59.1)	102 (67.5)	
**Encountered problems**				**0.049[4]**
None	265 (81.5)	135 (77.6)	130 (86.1)	
Any	60 (18.5)	39 (22.4)	21 (13.9)	
Financial	30 (9.2)	24 (13.8)	6 (4.0)	
Referral from one health facility to another	10 (3.1)	6 (3.4)	4 (2.6)	
Logistics	20 (6.2)	9 (5.2)	11 (7.3)	

1) p-values are calculated with Chi-Square tests excluded the row of 'data available'; 2) the proportion of data available is calculated, the proportion of the following subcategories are calculated with the available data as the denominator; 3) this p-value has been calculated with combining the subcategories 'very poor' and 'poor'; 4) this p-value has been calculated by comparing the categories 'none' and 'any'

Quality of care

Table 3 displays the means of the outcomes per interview question reflecting quality of care. Most of these given scores are high, i.e. above four. Lowest scores regard having an informed choice (total group 2.67, MNM 2.49 and controls 2.86) and being informed (total group 3.87, MNM 3.61 and controls 4.17), including being informed about the death of their baby (total

For peer review only

group 3.90, MNM 3.89 and controls 4.00). Mean differences between MNM and controls were statistically significant for some topics, such as having felt informed and perception of motivation of staff, but reflect small differences. One participant summarized her experience as follows:

"I am very satisfied because I am alive" – MNM participant, 28 years old, first pregnancy

Table 3: Means on a five-point scale (1=very poor, 2=poor, 3=not poor, not good, 4=good, 5=very good) and mean differences

	**Total**	**MNM**	**Controls**	
	**Mean (SD)**	**Mean (SD)**	**Mean (SD)**	**Mean difference (95% CI)**
Did you feel treated with respect?	4.54 (0.80)	4.50 (0.85)	4.58 (0.74)	-0.07 (-0.25–0.10)
Did you feel informed about treatments/ complications?	3.87 (1.54)	3.61 (1.62)	4.17 (1.40)	-0.55 (-0.89–0.22)
Did you feel you had an informed choice in the services you received?	2.67 (1.80)	2.49 (1.76)	2.86 (1.84)	-0.37 (-0.76–0.03)
Did you feel you had privacy?	4.47 (0.96)	4.36 (1.12)	4.59 (0.72)	-0.23 (-0.44–0.02)
Were you able to receive emotional support from relatives/friends?	4.47 (0.97)	4.51 (0.91)	4.42 (1.03)	0.09 (-0.12–0.30)
Did you feel you were provided with emotional support by the staff?	4.27 (1.18)	4.23 (1.20)	4.32 (1.16)	-0.09 (-0.35–0.17)
Did you feel that the staff was motivated and available?	4.41 (0.88)	4.32 (0.95)	4.52 (0.78)	-0.20 (-0.40–0.01)
Did you feel safe in the hospital?	4.65 (0.67)	4.63 (0.69)	4.66 (0.64)	-0.03 (-0.18–0.11)
Did you feel the health workers took the time for you?	4.51 (0.83)	4.47 (0.87)	4.56 (0.78)	-0.08 (-0.26–0.10)
Did you experience financial barriers to getting the right treatment?	4.59 (0.88)	4.47 (1.00)	4.74 (0.70)	-0.27 (-0.46–0.08)
Did you think the hospital had sufficient supplies to care for you? (Sanitation, medicine, equipment)	4.01 (0.99)	3.91 (0.97)	4.10 (1.02)	-0.17 (-0.39–0.05)
How do you feel about the way your baby was treated?	4.67 (0.77)	4.64 (0.87)	4.69 (0.70)	-0.05 (-0.28–0.18)
How do you feel about the overall experience of your admission?	4.33 (0.90)	4.24 (0.93)	4.42 (0.86)	-0.19 (-0.38–0.01)
In case baby died: how do you feel about the way you were informed on the death of your baby?	3.90 (1.54)	3.89 (1.56)	4.00 (1.48)	-0.11 (-1.08–0.85)
In case baby died: how do you feel about the way you, your baby and the baby's father were treated after your baby's death?	4.16 (1.39)	4.31 (1.26)	3.27 (1.85)	1.04 (0.16–1.91)

For peer review only

Would you recommend your sister/friend to deliver in the same place?	**Total N (%)**	**MNM N (%)**	**Controls N (%)**	
Yes	309 (95.1)	164 (94.3)	145 (96.0)	0.85 (0.57–1.26)
No	16 (4.9)	10 (5.7)	6 (4.0)	

As displayed in Table 4, a total of 70 interviewees (21.5%), comprising 43 MNM (24.7%), and 27 controls (17.9%), perceived the overall experience of the care during their admission as suboptimal. Most participants rated informed choice poorest, being scored as suboptimal by 57.5% of all participants. This is followed by sufficiency of supplies (28.3%), being informed in general (28.3%), and being informed in case of death of their baby (25.9%).

"If I had money I would go to a private hospital. The first doctor after the operation didn't tell me my baby died. … I felt ignored. …They didn't tell me anything. I am heartbroken here." – MNM participant, 31 years old, second pregnancy

Table 4: Suboptimal scores on quality of care reflecting absolute numbers and the proportion of the group of participants who answered with 1, 2 or 3. Odds ratios (OR) calculated for MNM vs. controls.

	**Total, N(%)**	**MNM, N(%)**	**Control, N(%)**	**OR (95% CI)**
**Total**	325 (100.0)	174 (53.5)	151 (46.5)	-
**Overall experience**	70 (21.5)	43 (24.7)	27 (17.9)	1.51 (0.88-2.59)
**Informed choice**	187 (57.5)	108 (62.1)	79 (52.3)	1.49 (0.96-2.32)
**Sufficient supplies**	104 (32.0)	61 (35.1)	43 (28.5)	1.36 (0.85-2.17)
**Information**	92 (28.3)	61 (35.1)	31 (20.5)	***2.09 (1.26-3.46)***
**Emtional support staff**	66 (20.3)	37 (21.3)	29 (19.2)	1.14 (0.66-1.96)
**Availability and motivation of staff**	57 (17.5)	38 (21.8)	19 (12.6)	***1.94 (1.07-3.54)***
**Time for patient**	43 (13.2)	22 (12.6)	21 (13.9)	0.90 (0.47-1.70)
**Financial barriers**	42 (12.9)	31 (17.8)	11 (7.3)	***2.76 (1.34-5.70)***
**Privacy**	41 (12.6)	29 (16.7)	12 (7.9)	***2.32 (1.14-4.72)***
**Emotional support family**	39 (12.0)	20 (11.5)	19 (12.6)	0.90 (0.46-1.76)
**Respect**	36 (11.1)	22 (12.6)	14 (9.3)	1.42 (0.70-2.88)
**Feeling of safety**	18 (5.5)	10 (5.7)	8 (5.3)	1.09 (0.42-2.84)
**Treatment of the baby**	10 (3.1)	4 (5.8)	6 (4.9)	0.57 (0.16-2.05)
		**Total N=73**	**Total N=12**	
**In case baby died: information**	22 (6.7)	20 (27.4)	2 (16.7)	***9.68 (2.22-42.11)***

**In case baby died: supportive treatment**	13 (4.0)	9 (12.3)	4 (33.3)	2.01 (0.60-6.65)

The odds of experiencing care as suboptimal were significantly higher for MNM concerning financial barriers in receiving high quality care (OR 2.76, 95% CI 1.34-5.70), privacy (OR 2.32, 95% CI 1.14-4.72), information provision (OR 2.09, 95% CI 1.26-3.46), and availability and motivation of staff (OR 1.94, 95% CI 1.07-3.54).

"They didn't tell me anything. I had no choice. I didn't feel extremely safe because some patients died. There is not enough medicine in the hospital and some check-ups needed to be done outside of the hospital, this costs a lot of money." – control participant, 23 years old, second pregnancy

"I don't know how my baby was treated. I haven't seen my baby yet at all. I don't know why, they told me I can't see him yet. I want to see him. I believe I will see him." – MNM participant, 29 years old, third pregnancy

Fig. 1 displays additional analysis on four domains, showing the proportion of participants who commented on the matter, among those who gave a high score and a suboptimal score, respectively. However, less than 10% of the participants provided any comments, except on informed choice where almost 20% of them did. Participants scoring the quality as good or very good were just as likely as participants scoring quality as suboptimal to give negative comments when asked to answer freely.

Figure 1: Good and suboptimal scores on domains of information provision, having an informed choice, having privacy and feeling to have been treated with respect, and the proportions of participants in both groups who commented on the index domains.

For peer review only

The following quotes further exemplify how participants who gave high quantitative scores did have critical comments on those topics:

"Someone said to me: stay where you are or I will beat you, while I was in labour. When I see that doctor now, I feel very uncomfortable." - MNM participant, 30 years old, second pregnancy > scored a 4 on 'respect'

"No privacy because even delivery was open and everyone could see" – MNM participant, 31 years old, seventh pregnancy > scored a 5 on 'privacy'

"Staff should change their attitude. There should be better rules/guidelines about how to treat patients. They should treat patients with more respect and listen to us. They should be more focused." – MNM participant, 24 years old, second pregnancy > scored a 4 on 'respect'

DISCUSSION

Main findings

In the journey to facility care, women who suffered from life-threatening complications and those who did not, equally had often experienced an extensive, not straight-forward process preceding the decision to go to the health care facility. Then, in reaching the facility, around one fifth of them, MNM significantly more often so, experienced barriers, such as a lack of money and transport. Once in the hospital, care was given quickly. Based on quantitatively scored questionnaires, the quality of care experienced was high. One fifth of all participants, MNM significantly more often than controls, had an overall suboptimal experience with informed choice and availability of supplies scored lowest. Our expectation was that participants would more often comment negatively if they had given lower scores but this did not show consistently. Generally, there was a positive quantitative assessment with critical comments expressed by a minority of the participants.

Interpretation

For peer review only

In our study population, MNM reported to have experienced financial barriers more often than the controls did. Previous work found that financial costs were a major obstacle in seeking non-emergency obstetric care, but not so much in emergency obstetric care,[13]. These results do not allow to distinguish whether MNM factually had higher costs or if they experienced a financial barrier and that led to developing complications. Additional research should be done to explore the nexus between economic factors and health-care seeking behaviour.

This study's methodology was mainly quantitative, but encouraged comments from the participants to enrich the numeric outcomes. Through calculations of averages we get a sense of experienced quality of care, but the valuable information for policy change really seems to come from the positive and negative extremes. The individual participants' comments reflected those extremes best, with their experiences of substandard care highlighting ample opportunities for improvement. This is most visible in their perception of quality of care which had a discrepancy between the quantitative and qualitative outcomes. We hypothesise that quantitative outcomes mainly reflect the norms in the health care system and broader society, with people adjusting their standards to what is currently available to them. Even so, qualitative data did shed a bright light on personal needs and preferences deviating from the current norm. The norm might be what it is, but that does not mean that the patient, who is depending on the health care system, finds the norm acceptable.

For example, the vast majority scored 'privacy' as good or very good, whilst one in every 20 participants shared how they had to share beds, there was a lack of curtains and physical examinations were executed without shielding. The quotes reproduced in the article illustrate stories on poor treatment during hospitalization that are similar to those of other women around the world,[14-17], whose expectations of smooth access, privacy, adequate provision of information, attitude and availability of supplies reflect essential elements of high-quality maternal care as standardized by the WHO,[8]. Dissatisfaction on not meeting these expectations might not be reflected directly given the propensity to normative answers in most,

For peer review only

but can negatively impact future service utilization and, consequently, maternal health outcomes.

Our study advocates to use both quantitative and qualitative methods to get a genuine impression of patients' perspectives and experiences. Theories in the domains of social and economic welfare have argued the potential value of patients' perspectives in health research,[18], claiming how a patient's direct testimony of the experienced illness and received health care might be too positive when compared to the public's supposedly more objective opinion. The concept of adaptive preferences explains how health care, which is formally perceived as substandard, might be perceived as sufficient or good by the receiving individual at that time. We argue, though, partly in line with Nussbaum's philosophy on this, that any patient's testimony, at any time and any place, is valid in and of itself,[18,19]. Furthermore, following a human rights-based approach to maternal health care, we believe it to be quintessential, in the pursue of universal high-quality care for all, to include women and their communities in maternal health research and policy making,[20].

Strengths and limitations

This study involved a wide range and large number of patients from the maternal population in Zanzibar's main hospital, which deals with a high volume of non- and mildly complicated cases as well as with, in its role of referral hospital, the most complicated cases. Nevertheless, it is not a complete representation of the patient population, leading to selection bias. We could not include women who had not reached the hospital, including those who may suffer most from financial barriers. The same applies to the exclusion of patients who were not reachable by mobile phone, which possibly excluded those suffering more from poverty and possibly having less influence in their households and in decision-making processes regarding health. Additionally, conduction of the (majority of) interviews in the hospital made the outcomes prone to information bias, discouraging people to criticize the facility they were still in and, sometimes,

For peer review only

depending on. Furthermore, we cannot exclude moderator and reporter bias, which we tried to mitigate through a well-defined methodology and interview structure.

In the MNM group, experiences might have been more positive because of having received comprehensive care when it was needed the most, ultimately leading to them surviving life-threatening illness. Equally, experiences might have been more negative because of a more extensive exposure to the health care system and its flaws. Amongst the not so complicated cases, experiences might have been more positive because of an easier process, although these patients might have felt less surrendered to/dependent of the health care system and, therefore, freer to be critical.

Within the group of participants who commented critically beyond the set interview questions, we reached outcome saturation, thus trusting their comments to reflect a daily experienced reality and consequently providing policy makers with starting points for health care improvement.

Methodological challenges led to a percentage of the MNM during the study period to not have been interviewed and for a lower number of control participants to have been included. The final large number of included participants, however, makes us believe that this has not led to selection bias.

CONCLUSION

This study assessed the perception of patients on their journey to and during maternal care in Zanzibar's main health facility. Our outcomes reflect first-hand testimonies, informing us that most patients seeking care in MMH during pregnancy and childbirth seek care on time, access MMH quickly and are treated on time. A significant minority of them, though, experiences barriers, mostly financial, in reaching care, with MNM more so than controls. Quantitatively assessed, quality of care is scored high and is similar between MNM and controls. Simultaneously, a group of participants shared insightful critical perceptions. There seems to

For peer review only

be an opportunity to distinguish between normative and personal perceptions. To continue to conduct patient-centred and setting-specific maternal health research in the pursue of improved maternal well-being, we suggest to combine quantitative and qualitative methodologies.

REFERENCES

1. Alkema L, Chou D, Hogan D, et al. Global, regional, and national levels and trends in maternal mortality between 1990 and 2015, with scenario-based projections to 2030: a systematic analysis by the UN Maternal Mortality Estimation Inter-Agency Group. *Lancet* 2016;387(10017):462-74 doi: 10.1016/S0140-6736(15)00838-7.
2. Garbysch S, Nesbitt RC, Schoeps A, et al. Does facility birth reduce maternal and perinatal mortality in Brong Ahafo, Ghana? A secondary analysis using data on 119 244 pregnancies from two cluster-randomised controlled trials. *Lancet Glob Health* 2019;7:e1074–87.
3. World Health Organization. Quality, equity, dignity: the network to improve quality of care for maternal, newborn and child health – strategic objectives. Geneva: WHO 2018.
4. Bohren MA, Mehrtash H, Fawole B, et al. How women are treated during facility-based childbirth in four countries: a cross-sectional study with labour observations and community-based surveys. *Lancet* 2019;394:1750–1763.
5. Solnes Miltenburg A, van Pelt S, Meguid T,et al. Disrespect and abuse in maternity care: individual consequences of structural violence. *Reprod Health Matters* 2018;26(53):88-106 doi:10.1080/09688080.2018.1502023.
6. Ministry of Health, Revolutionary Government of Zanzibar. Zanzibar Annual Health Bulletin. Zanzibar, United Republic of Tanzania 2016.
7. Herklots T, van Acht L, Khamis RS, et al. Validity of WHO's near-miss approach in a high maternal mortality setting. *PLoS ONE* 2019;14(5):e0217135 doi: 10.1371/journal.pone.0217135.

For peer review only

8. World Health Organization. Standards for improving quality of maternal and newborn care in health facilities. Geneva: WHO 2016.

9. Fakih B, Azzah Nofly AS, Ali AO, et al. The status of maternal and newborn health care services in Zanzibar. *BMC Pregnancy and Childbirth*. 2016;16:134 doi: 10.1186/s12884-016-0928-6.

10. Gon G, Ali SM, Towriss C, et al. Unpacking the enabling factors for hand, cord and birth-surface hygiene in Zanzibar maternity units. *Health Policy Plan* 2017;32:1220-1228 doi: 10.1093/heapol/czx081.

11. World Medical Association Declaration of Helsinki: ethical principles for medical research involving human subjects. *JAMA* 2013;310(20):2191-2194 doi:10.1001/jama.2013.281053.

12. Herklots T, van Acht L, Meguid T, et al. Severe maternal morbidity in Zanzibar's referral hospital: Measuring the impact of in-hospital care. *PLoS One* 2017;12(8):e0181470 doi: 10.1371/journal.pone.0181470.

13. Norhayati MN, Surianti S, Hussain Nik Hazlina N. Metasynthesis: Experiences of women with severe maternal morbidity and their perception of the quality of health care. *PLoS One* 2015;10(7): e0130452 doi: 10.1371/journal.pone.0130452.

14. Tunçalp Ö, Hindin MJ, Adu-Bonsaffoh K, et al. Listening to women's voices: The quality of care of women experiencing severe maternal morbidity, in Accra, Ghana. *PLoS One* 2012;7(8): e44536 doi: 10.1371/journal.pone.0044536.

15. Rööst M, Jonsson C, Liljestrand J, et al. Social differentiation and embodied dispositions: a qualitative study of maternal care-seeking behaviour for near-miss morbidity in Bolivia. *Reprod Health* 2009;29;6:13 doi: 10.1186/1742-4755-6-13.

16. Weeks A, Lavender T, Nazziwa E, et al. Personal accounts of 'near-miss' maternal mortalities in Kampala, Uganda. *BJOG* 2005;112(9):1302-7.

17. Norhayati MN, Nik Hazlina NH, Ab Razak A, et al. The experiences of women with maternal near miss and their perception of quality of care in Kelantan, Malaysia: a

For peer review only

qualitative study. *BMC Pregnancy and Childbirth* 2017;17:189 doi: 10.1186/s12884-017-1377-6.

18. Nussbaum M. Symposium on Amartya Sen's philosophy: Adaptive preferences and women's options. *Economics & Philosophy* 2001; 17(1):67-88 doi: 10.1017/S0266267101000153.

19. Mitchell P. Adaptive Preferences, Adapted Preferences. *Mind* 2018; 127(508):1003-1025 doi: 10.1093/mind/fzy020.

20. Freedman LP. Using human rights in maternal mortality: from analysis to strategy. *Int J Gynaecol Obstet* 2001;75:51-60.

FIGURE LEGEND

Figure 1. Legend: top bar left side= suboptimal score with no comment; top bar right side= suboptimal score with comment; bottom bar left side= good score with no comment; bottom bar right side= good score with comment

SUPPLEMENTARY FILES

Table 1: Markers for organ dysfunction, identifying cases in life-threatening condition

Table 2: Interview outline

AUTHOR CONTRIBUTIONSHIP

TH, TM and BJ conceptualized the study. TH, KMS, MR and LD conducted the majority of the data collection and were responsible for data integrity. TH, SC and MPHK performed data analysis. TH and TM supervised the research on-site. BJ and AF supervised the research off-site. TH drafted the manuscript. All authors revised multiple versions of the manuscript. All authors have read and approved the final version of the manuscript.

For peer review only

COMPETING INTERESTS

None declared.

ETHICAL APPROVAL

Ethical approval was obtained from Zanzibar's Medical Research and Ethics Committee under protocol reference number ZAMREC/0002/JUN/17.

DATA SHARING STATEMENT

All data relevant to the study are included in the article or uploaded as supplementary information. Deidentified participant data is available from the study's first author (ORCID: https://orcid.org/0000-0003-4513-0063). Reuse is solely permitted after reasonable request with permission from the involved medical ethical committee, the hospital's administration and the study's principal investigator. The study protocol is available upon reasonable request.

FUNDING

No specific funding was received for this study

ACKNOWLEDGEMENTS

We thank Zuwena Ali Rashid, Vreni Bron, Suhaila Salum Yussuf, Sigrid Hilgen, Marloes Plender, Lisa Trommelen, Jenna Uijt de Haag, Jasmijn Rake, Jasmijn Broerze, Ibrahim Shaban, Elleke van der Meij, Eline Veenstra and Claire Bausch for their enthusiastic, tireless contributions to the data collection. We thank all health workers of Mnazi Mmoja Hospital's

For peer review only

491 departments of Obstetrics & Gynaecology, Internal Medicine, Surgery and Intensive Care for facilitating and supporting participant inclusion and data collection. We thank the administration of Mnazi Mmoja Hospital for facilitating and supporting this study. We are deeply grateful to all the participants for opening up and sharing with us their opinions and ideas.

For peer review only

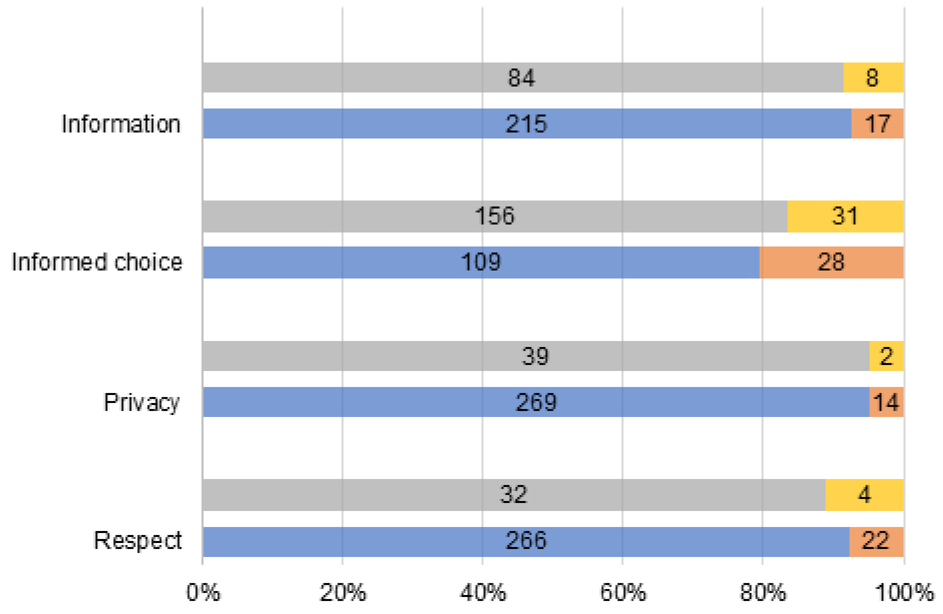

87x59mm (144 x 144 DPI)

Table 1: Markers for organ dysfunction, identifying cases in life-threatening condition

Type of organ dysfunction	Markers (clinical signs, laboratory markers and management actions)
**Cardiovascular**	Shock[1], use of continuous vasoactive drugs, cardiac arrest, cardio-pulmonary resuscitation, severe hypoperfusion (lactate >5 mmol/L or >45mg/dL)[2] or severe acidosis (pH <7.1)[2]
**Respiratory**	Acute cyanosis, gasping[3], severe tachypnea (respiratory rate >40bpm), severe bradypnea (respiratory rate <6bpm), severe hypoxemia (PAO2/FiO2 <200mmHg[2] or O2 saturation <90% for ≥60min) or intubation and ventilation not related to anaesthesia
**Renal**	Oliguria[4] non-responsive to fluids or diuretics, dialysis for acute renal failure[5] or severe acute azotaemia (creatinine ≥300umol/ml or ≥3.5mg/dL)
**Coagulation / haematological**	Clotting failure[6], use of continuous vasoactive drugs[7], massive transfusion of blood or red cells (≥5 units)[8] or severe acute thrombocytopenia (<50,000 platelets/ml)
**Hepatic**	Jaundice in the presence of pre-eclampsia[9], severe acute hyperbilirubinemia (bilirubin >100umol/L or >6.0mg/dL)
**Neurological**	Prolonged unconsciousness (lasting >12 hours)/coma[10], stroke[11], status epilepticus[12], uncontrollable fits/total paralysis
**Uterine**	Hysterectomy following haemorrhage or infection

1) Shock is a persistent severe hypotension, defined as a systolic blood pressure <90 mmHg for ≥60 minutes with a pulse rate at least 120 despite aggressive fluid replacement (>2l)
2) Laboratory test or management intervention that is not available at MMH
3) Gasping is a terminal respiratory pattern and the breath is convulsively and audibly caught
4) Oliguria is defined as a urinary output <30 ml/hr for 4 hours or <400 ml/24 hr
5) Dialysis services have been available in MMH since 26 May 2017
6) Clotting failure can be assessed by the bedside clotting test or absence of clotting from the intravenous site after 7–10 minutes
7) For instance, continuous use of any dose of dopamine, epinephrine or norepinephrine
8) In MMH extended to include all types of blood products and cases in which 5 or more units were requested but not given due to shortage
9) Pre-eclampsia is defined as the presence of hypertension associated with proteinuria. Hypertension is defined as a blood pressure of at least 140 mmHg (systolic) or at least 90 mmHg (diastolic) on at least two occasions and at least 4–6 h apart after the 20th week of gestation in women known to be normotensive beforehand. In MMH, proteinuria is defined as ≥2+ protein on dipstick.
10) Loss of consciousness is a profound alteration of mental state that involves complete or near-complete lack of responsiveness to external stimuli. It is defined as a Coma Glasgow Scale <10 (moderate or severe coma).
11) Stroke is a neurological deficit of cerebrovascular cause that persists beyond 24 hours or is interrupted by death within 24 hours
12) Condition in which the brain is in a state of continuous seizure

For peer review only

Table 2: Interview outline

PATIENT INTERVIEW – BEFORE DISCHARGE (English version)										**NO.:**
**Topic**	**Questions**									
**1) Patient characteristics**	Age:	Level of education (primary, secondary, tertiary):								
	Gravidity/Parity:	Marital status (first-, second-, third- or fourth wife, divorced, widow, single):								
	Number of living children:	Perceived wealth: Very poor – Poor – Average – Rich – Very Rich								
	Number of children of school-going age attending school:	Occupation: Paid work – Self-employed – Non-paid work – Student – Housework – Unemployed for health reason – Unemployed for other reason – Other, …………………								
	Smoking?	Alcohol?	Drugs?							
**2) Family Planning**	Pregnancy planned?	If unplanned, wanted?	Did you have access to contraceptives?							
			**1**	**2**	**3**	**4**	**5**	**na**		
**3) Antenatal Care (ANC)**	**How did you experience the access of ANC?** Scale: 1=impossible, 2=difficult, 3=not simple, not difficult, 4=simple, 5= very simple, na=not applicable									
	**How do you feel about your overall experience in ANC?** Scale: 1=very dissatisfied, 2=dissatisfied, 3=neither satisfied, nor dissatisfied, 4=satisfied, 5= very satisfied, na=not applicable									
	**Did you consult others for treatment during your pregnancy such as traditional birth attendants or others?**									
	a. No									
	b. Yes, traditional birth attendant									
	c. Yes, other									
	**Comments:**									
**4) Access to Health Facility**	**Where did you plan to deliver?**									
	a. Home									
	b. Health center									
	c. Other hospital									
	d. MMH									
	**What was the main motivation for delivering there?**									
	**Who accompanied you to the health facility and took care of you during hospitalization?**									
	a. Female family member									
	b. Husband									
	c. Friend									
	d. Other									
	**Who took care of your children during your hospitalization?**									
	a. Female family member									
	b. Husband									
	c. Friend									
	d. Other									

For peer review only

		1	2	3	4	5	na
	**How did you experience the access to the health facility?** Scale: 1=very poor, 2=poor, 3=not good, not poor, 4=good, 5= very good, na=not applicable						
	**If you encountered problems, what kind of problems?**						
	a. Financial						
	b. Referral						
	c. Logistic						
	d. No problems						
	**How did you come here and how long did that take?**						
	**Comments:**						
**5) Perception on Development of Disease/ Complications**	**How much time passed between when you started feeling less well and you sought medical help?**						
	**How much time passed between when you arrived in the hospital/you started feeling less well in the hospital and the moment a health worker came to see/question/examine you?**						
	**Did you recognise that you started to feel worse? If so, how did you feel and what did you think?**						
		1	2	3	4	5	na
	**Did you have knowledge about your disease/ complication(s) before you suffered from it yourself? If so, how?** Scale: 1=not at all, 2= little, 3= moderate amount, 4=good knowledge, 5=extreme good knowledge, na=not applicable						
	**Comments**						
		1	2	3	4	5	na
**6) Perceived Quality of Care during Hospitalization**	**Did you feel treated with respect?** Scale: 1=not at all, 2=a little, 3=a moderate amount, 4=very much, 5=extremely, na=not applicable						
	**Did you feel informed about treatments/ complications?** Scale: 1=not at all, 2=a little, 3=a moderate amount, 4=very much, 5=extremely, na=not applicable						
	**Did you feel you had an informed choice in the services you received?** Scale: 1=not at all, 2=a little, 3=a moderate amount, 4=very much, 5=extremely, na=not applicable						
	**Did you feel you had privacy?** Scale: 1=not at all, 2=a little, 3=a moderate amount, 4=very much, 5=extremely, na=not applicable						
	**Were you able to receive emotional support from relatives/friends?** Scale: 1=not at all, 2=a little, 3=a moderate amount, 4=very much, 5=extremely, na=not applicable						
	**Did you feel you were provided with emotional support by the staff?** Scale: 1=not at all, 2=a little, 3=a moderate amount, 4=very much, 5=extremely, na=not applicable						

For peer review only

		1	2	3	4	5	na
	**Did you feel that the staff was motivated and available?** Scale: 1=not at all, 2=a little, 3=a moderate amount, 4=very much, 5=extremely, na=not applicable						
	**Did you feel safe in the hospital?** Scale: 1=not at all, 2=a little, 3=a moderate amount, 4=very much, 5=extremely, na=not applicable						
	**Did you feel the health workers took time for you?** Scale: 1=not at all, 2=a little, 3=a moderate amount, 4=very much, 5=extremely, na=not applicable						
	**Did you experience financial barriers to getting the right treatment?** Scale: 1=extremely, 2=very much, 3=a moderate amount, 4=a little, 5=not at all, na=not applicable						
	**Did you think the hospital had sufficient supplies to care for you? (Sanitation, medicine, equipment)** Scale: 1=not at all, 2=a little, 3=a moderate amount, 4=very much, 5=extremely, na=not applicable						
	**How do you feel about the way your baby was treated?** Scale: 1=very dissatisfied, 2=dissatisfied, 3=neither satisfied, nor dissatisfied, 4=satisfied, 5= very satisfied, na=not applicable						
	**How do you feel about the overall experience of your admission?** Scale: 1=very dissatisfied, 2=dissatisfied, 3=neither satisfied, nor dissatisfied, 4=satisfied, 5= very satisfied, na=not applicable						
	If your baby died:						
		**1**	**2**	**3**	**4**	**5**	**na**
	**How do you feel about the way you were informed on the death of your baby?** Scale: 1=very dissatisfied, 2=dissatisfied, 3=neither satisfied, nor dissatisfied, 4=satisfied, 5= very satisfied, na=not applicable						
	**How do you feel about the way you, your baby and the baby's father were treated after your baby's death?** Scale: 1=very dissatisfied, 2=dissatisfied, 3=neither satisfied, nor dissatisfied, 4=satisfied, 5= very satisfied, na=not applicable						
	**Comments:**						
	**SCALE** 1=an extreme impact, 2= a high impact, 3=a moderate impact, 4= little impact, 5= no impact at all, na=not applicable	**1**	**2**	**3**	**4**	**5**	**na**
**7) Perspective on Impact of Hospitalization**	**Impact on your position within your close family**						
	**Impact on your position within extended family/community**						
	**Financial and economic impact of hospitalization**						
	**Impact on educational prospects your children**						
	**Comments:**						
**8) State upon Discharge**	**Do you feel recovered at discharge?** Yes/No						
	**Do you feel able to resume your daily activities?** Yes/No						

**9) Recommend**	**Would you recommend your sister to deliver in the same place?** Yes/No
**10) Suggestions**	**Do you have any suggestions to improve the care for you and your baby in the future? What would you like to see different?**

For peer review only

SRQR Checklist:

S1 – title > see title, line 1-3

S2 – abstract > see abstract, page 2

Introduction

S3 – problem formulation > line 77-112

S4 – purpose or research question > line 118-122

Methods

S5 – qualitative approach and research paradigm > line 126, 148-151

S6 – researcher characteristics and reflexivity > 140-141

S7 – context > line 99-112

S8 – sampling strategy > line 127-32

S9 – ethical issues pertaining to human subjects > line 133-135

S10 – data collection methods > line 138-141

S11 – data collection instruments and technologies > line 138-141

S12 – units of study > line 141-148, line 175-178

S13 – data processing > line 152

S14 – data analysis > line 152-161, line 170-172

S15 – techniques to enhance trustworthiness > line 140-141, line 162-169

Results/findings

S16 – synthesis and interpretation > line 181-301

S17 – links to empirical data > throughout line 181-301

Discussion

S18 – integration with prior work, implications, transferability, and contribution(s) to the field > line 321-364

S19 – limitations > line 367-393

Other

S20 – conflicts of interest > line 483-484

S21 – funding > line 493-494

For peer review only

BMJ Open

The journey to facility birth in Zanzibar: a questionnaire-based cohort study of patients’ perspectives on preparedness, access and quality of care

Journal:	*BMJ Open*
Manuscript ID	bmjopen-2020-040381.R2
Article Type:	Original research
Date Submitted by the Author:	11-Nov-2020
Complete List of Authors:	Herklots, Tanneke; Erasmus MC, Obstetrics & Gynaecology<br>D'haene, Lara; Hospital Doctor Jose Molina Orosa, Emergency Medicine<br>Mbarouk, Khairat; Mnazi Mmoja Referral Hospital, Obstetrics & Gynaecology<br>Rajhy, Mubina; Mnazi Mmoja Referral Hospital, Obstetrics & Gynaecology<br>Couperus, Simone; University of Utrecht Faculty of Medicine, Obstetrics &Gynaecology<br>Meguid, Tarek ; Village Health Works<br>Franx, Arie; Erasmus MC, Obstetrics & Gynaecology<br>Koster, Maria PH; Erasmus MC, Obstetrics & Gynaecology<br>Jacod, Benoit; Onze Lieve Vrouwe Gasthuis, Obstetrics & Gynaecology
**Primary Subject Heading**:	Obstetrics and gynaecology
Secondary Subject Heading:	Global health, Patient-centred medicine, Reproductive medicine
Keywords:	Maternal medicine < OBSTETRICS, Quality in health care < HEALTH SERVICES ADMINISTRATION & MANAGEMENT, Organisation of health services < HEALTH SERVICES ADMINISTRATION & MANAGEMENT, REPRODUCTIVE MEDICINE

SCHOLARONE™
Manuscripts

I, the Submitting Author has the right to grant and does grant on behalf of all authors of the Work (as defined in the below author licence), an exclusive licence and/or a non-exclusive licence for contributions from authors who are: i) UK Crown employees; ii) where BMJ has agreed a CC-BY licence shall apply, and/or iii) in accordance with the terms applicable for US Federal Government officers or employees acting as part of their official duties; on a worldwide, perpetual, irrevocable, royalty-free basis to BMJ Publishing Group Ltd ("BMJ") its licensees and where the relevant Journal is co-owned by BMJ to the co-owners of the Journal, to publish the Work in this journal and any other BMJ products and to exploit all rights, as set out in our licence.

The Submitting Author accepts and understands that any supply made under these terms is made by BMJ to the Submitting Author unless you are acting as an employee on behalf of your employer or a postgraduate student of an affiliated institution which is paying any applicable article publishing charge ("APC") for Open Access articles. Where the Submitting Author wishes to make the Work available on an Open Access basis (and intends to pay the relevant APC), the terms of reuse of such Open Access shall be governed by a Creative Commons licence – details of these licences and which Creative Commons licence will apply to this Work are set out in our licence referred to above.

Other than as permitted in any relevant BMJ Author's Self Archiving Policies, I confirm this Work has not been accepted for publication elsewhere, is not being considered for publication elsewhere and does not duplicate material already published. I confirm all authors consent to publication of this Work and authorise the granting of this licence.

For peer review only

The journey to facility birth in Zanzibar: a questionnaire-based cohort study of patients' perspectives on preparedness, access and quality of care

Tanneke Herklots[1*], Lara D'haene[2], Khairat Mbarouk Said[3], Mubina Rajhy[3], Simone Couperus[4], Tarek Meguid[5], Arie Franx[1], Maria PH Koster[1], Benoit Jacod[6]

1. Department of Obstetrics & Gynaecology, Erasmus MC, University Medical Centre Rotterdam, Rotterdam, The Netherlands
2. Department of Emergency Medicine, Hospital Doctor Jose Molina Orosa, Lanzarote, Spain
3. Department of Obstetrics & Gynaecology, Mnazi Mmoja Hospital, Zanzibar, United Republic of Tanzania
4. Department of Obstetrics & Gynaecology, University Medical Centre Utrecht, Utrecht, The Netherlands
5. Village Health Works, Kigutu, Burundi
6. Department of Obstetrics & Gynaecology, Onze Lieve Vrouw Gasthuis, Amsterdam, The Netherlands

*) Corresponding author: full name: Tanneke Herklots; postal address: Westerkade 20A, 9718AS, Groningen, The Netherlands; e-mail: tannekeherklots@gmail.com

Word count abstract: 299

Word count full text: 3756

For peer review only

ABSTRACT

Introduction

Tackling substandard maternity care in health facilities requires engaging women's perspectives in strategies to improve outcomes. This study aims to provide insights in the perspectives of women with severe maternal morbidity on preparedness, access and quality of care in Zanzibar's referral hospital.

Methods

In a prospective cohort from April 2017 until December 2018, we performed semi-structured interviews with women who experienced maternal near-miss complications and matched controls. These focused on sociodemographic and obstetric characteristics, perceived accessibility to and quality of facility care with 15 domains, scored on a one-to-five scale. Participants' comments and answers to open questions were employed to illustrate quantitative outcomes. Zanzibar's Medical Research and Ethics Committee approved the study (ZAMREC/0002/JUN/17).

Results

We included 174 cases and 151 controls. Compared to controls, patients with a near-miss had less formal education ($p=0.049$), perceived their wealth as poor ($p=0.002$), and had a stillbirth more often ($p<0.001$). Many experienced a delay in deciding to seek care. More than controls, near-miss patients experienced barriers in reaching care ($p=0.049$), often of financial nature (13.8% vs. 4.0%). Quality of care was perceived as high, with means above three out of five, in 14 out of 15 domains. One fifth had an overall suboptimal experience, mostly regarding informed choice and supplies availability. Additional comments were expressed by a minority of participants.

Conclusion

Most patients promptly sought, accessed and received maternity care in Zanzibar's referral hospital. A minority experienced barriers, mostly financial, in reaching care, and more so among patients with near-miss complications. Quality of facility care was generally highly

For peer review only

rated. However, some reported insightful critical perceptions. This study highlights the impact of sociodemographic differences on health, the value of involving patients in decisions regarding maternity care, and the need to ensure availability of medical supplies, all which will contribute to improved maternal well-being.

STRENGTHS AND LIMITATIONS OF THIS STUDY

- The study involved a wide range and large number of maternal patients in Zanzibar's main hospital
- The given comments reached outcome saturation, for which we trust them to experienced reality, thus being valid starting points for healthcare improvement to provide to policy makers
- This study displays how facility maternity care is perceived by the patients themselves, and does so in an extensive, profound and both quantitative and qualitative manner
- Because we could not include women who had not reached the hospital, nor patients who were not in possession of a mobile phone, the study had a selection bias potentially at the cost of those suffering more from poverty
- There might have been a certain level of information bias because the (majority of) interviews was conducted in the hospital, potentially discouraging people to criticize the facility they were at

For peer review only

INTRODUCTION

Low-income countries carry a disproportionately large burden of maternal morbidity and mortality. Efforts have gone into ensuring availability of essential interventions, promoting facility deliveries and skilled birth attendance,[1]. Unfortunately, despite a widespread increase in facility deliveries, the improvement in pregnancy outcomes has been slower than expected. This might be because women and their families are not prepared in time to seek facility care or face limitations in accessing facility care, but there seems to be a causal role for the low quality of care provided in many facilities,[2]. Low quality of care negatively affects the willingness to seek facility care, either in a future pregnancy or of other pregnant women in the community. Poor quality of care is not limited to but includes maltreatment during childbirth, which has been found to be common in many low-income settings,[3-5]. Tackling substandard maternity care requires incorporating the perspectives of women in designing strategies to improve maternal and child outcomes.

A specific group of women within the health care system are those who experience life-threatening complications during pregnancy and childbirth, so-called maternal near-miss (MNM) complications, but survive. Maternal deaths are an often-used proxy for assessing the quality of care but in the group of all child-bearing women, represent an absolute small number. Women who had an MNM (frow now on referred to as MNM) represent a larger proportion, whilst still being a group small enough to accommodate in-depth assessments of their multi-facetted experiences in the health care system.

In Zanzibar, Tanzania, around half of the deliveries take place at home and half in a health facility,[6]. Of the latter, around 30% take place in the region's main public referral health facility: Mnazi Mmoja Hospital (MMH). The maternal mortality rate (MMR) at MMH is around 400 per 100,000 livebirths,[7]. Multiple quality of care assessments have been performed at Tanzanian health care facilities guided by the World Health Organization's (WHO) Standard for Care,[8]. In 2012, a large unmet need for basic and comprehensive emergency obstetric and neonatal care was found throughout Zanzibar, including shortages of staff and supplies,

For peer review only

and an insufficient geographical access,[9]. In 2016, an assessment in Zanzibar showed that five main governmental hospitals, including MMH, were overall not conducive for high-quality maternal and child health care provision (Meguid T, Mrisho Haji F, Herklots T, Rajhy M, Kassim NI. Conduciveness for High Quality of Maternal- and Child Health Care in Hospitals in Zanzibar. 2016). In 2017, an assessment of 37 maternity units in Zanzibar showed how more than half of those had challenges in providing hand and surface hygiene,[10].

This objective of this study is to explore the experiences of women with and without near-miss complications regarding their preparedness for, access to and quality of facility care in Zanzibar's main hospital. Understanding maternity care with its qualities and flaws from the patients' perspectives can inform local and regional maternal health policies and research agendas.

METHODS

From April 2017 until December 2018, we conducted a prospective cohort study at the department of Obstetrics & Gynaecology of MMH. We performed semi-structured interviews with women who experienced an MNM and control participants. MNM were identified during the department's twice daily meetings by a locally adapted and validated version of the WHO near-miss approach (for criteria see Supplementary Files, Table 1),[7]. Controls, matched on a 1:1 ratio, did not have a severely complicated pregnancy and had a similar date of admission (up to three days before or after the MNM's admission date), similar mode of delivery (vaginal, by caesarean section or instrumentally-assisted) and similar gestational age (first or second trimester or third trimester pre-term or term) as MNM.

This research was approved by Zanzibar's Medical Research and Ethics Committee (ZAMREC/0002/JUN/17) and conducted in line with the principles of the Declaration of Helsinki,[11]. In addition to providing informed consent, inclusion criteria were age of 18 years or above, no severe pre-diagnosed psychiatric disorders, residence on Unguja (Zanzibar's main island on which MMH is located) and to be reachable by mobile phone. Informed consent

of all participants was obtained in writing or verbally, in case of illiteracy. An interview was performed before discharge or, in case of logistic restrictions, within one week of discharge at the participant's home. The interviews were performed by a researcher and a mediator/translator, both not involved in the clinical care of the woman. The interview comprised the following: sociodemographic characteristics (age, self-reported level of formal education, type of occupation, marital status, perceived wealth), obstetric characteristics (parity – with a grand multiparity defined as a parity above 4, singleton or multiple pregnancy, gestational age, planned location for delivery and pregnancy outcome being abortion, live birth or still birth), utilization and opinion of antenatal care (ANC) services (not included in this publication), perception on the accessibility of facility care and perception on the quality of facility care (see Supplementary Files, Table 2, for the interview outline). We decided to combine quantitative and qualitative outcomes. Employing quantitative measurements of experiences around child birth and satisfaction with health care are in line with recent efforts to design validated standard outcome measurements sets, including patient-reported outcomes, such as the Pregnancy and Childbirth set of the International Consortium for Health Outcomes Measurement (ICHOM),[12]. A quantitative approach allows for easier comparison between categories, across settings and over time. While discussing those experiences on accessibility to and quality of care, the interviewer requested interviewees' comments in addition to the quantitative scores, which yielded a wealth of quotes. >

SPSS Statistics (version 25) and OpenEpi (version 3.01) were used for statistical analysis. Numeric outcomes were categorized similarly as in previous studies in this setting: age younger than 20 years, 20 to 35 years and older than 35 years; and parity zero, one to four or higher than four,[7,13]. Baseline characteristics were compared using Pearson's chi-square tests and, in case of small sample sizes, Fisher's exact test. Regarding quality of care, the interview outcomes were on a five-point scale, from which means per question were calculated and compared between MNM and controls using Mann-Whitney-U-tests. Suboptimal care was defined as a score of 3 or lower. Proportions of participants grading the assessed element as

For peer review only

suboptimal were reflected in percentages. Odds ratios (OR) with 95% confidence intervals (CI) were calculated to compare between MNM and controls, to assess if having had a pregnancy with a life-threatening complication affects quality of care perception. We observed a discrepancy between the fact that the majority of patients perceived quality of care as good or very good and the fact that give comments most often had a negative connotation. To investigate this discrepancy further, we compared the frequency of negative comments in participants having scored as positive or as suboptimal on the domain to which the comments pertain. We deemed the number of participants giving comments to be sufficiently high to perform this analysis on four domains: information, informed choice, respect and privacy.

A p-value below 0.05 was considered statistically significant. Quotations taken from participants' comments and answers to open questions were employed to illustrate the quantitative outcomes.

Patient and public involvement

The lack of patient's perspectives in maternal health research in our setting motivated our research questions and methodology. Patients and public were not directly involved in the design of the study, nor in the plans for dissemination. The flexible construct of the interviews did allow the patient to decide on which topics she wanted to elaborate. Dissemination of the study outcomes will, next to publication in an open access scientific journal, take place in the study setting, to the index hospital and to Zanzibar's Ministry of Health. We hope this study can inspire the local research agenda, motivating patient involvement.

RESULTS

During the study period, 269 patients were identified as MNM, of whom 174 (64.7%) were interviewed. Reasons for no study participation were patients' personal reasons, meeting one of the exclusion criteria or an unexpected early discharge home. A total of 151 control participants was interviewed.

For peer review only

Participants' characteristics

Baseline and outcome characteristics are displayed in Table 1. MNM and controls were comparable at baseline regarding age, occupation and marital status. MNM were significantly more likely to have completed primary education only (29.9% vs. 19.2%, p=0.05), more often perceived their own wealth as very poor or poor (19.0% vs. 7.3%, p=0.002), were less often nulliparous (32.2% vs. 50.3%, p=0.002), and more often grand multiparous (13.9% vs. 6.0%, p=0.002). Of the MNM pregnancies, 42.9% ended in a stillbirth compared to 5.4% of control pregnancies (p<0.001). Nearly two-thirds of all participants had initially planned to deliver at MMH, controls significantly more so than MNM (total 66.5%; 58.6% MNM vs. 75.5% controls, p=0.004).

Table 1: Baseline and outcome characteristics

	Total N (%)	MNM N (%)	Controls N (%)	p-value
**Total**	325 (100)	174 (53.5)	151 (46.5)	
**Age**				0.25
Data available[1]	324 (99.7)	174 (100)	150 (99.3)	
<20 years	16 (4.9)	11 (6.3)	5 (3.3)	
20-35 years	267 (82.4)	138 (79.3)	129 (86.0)	
>35 years	41 (12.7)	25 (14.4)	16 (10.7)	
**Parity**				**0.002**
Data available[1]	324 (99.7)	173 (99.4)	151 (100)	
0 (nulliparous)	132 (40.7)	56 (32.4)	76 (50.3)	
1-4 (multiparous)	159 (49.1)	93 (53.8)	66 (43.7)	
5 or more (grand multiparous)	33 (10.2)	24 (13.9)	9 (6.0)	
**Formal education level**				**0.049**
No formal education	24 (7.4)	15 (8.6)	9 (6.0)	
Primary	81 (24.0)	52 (29.9)	29 (19.2)	
Secondary	194 (59.7)	97 (55.7)	97 (64.2)	
Higher	26 (8.0)	10 (5.7)	16 (10.6)	
**Occupation**				0.80
Paid work or self-employed	148 (45.5)	76 (43.7)	72 (47.7)	
Housework	163 (50.2)	91 (52.3)	72 (47.7)	
Non-paid work or student	9 (2.8)	5 (2.9)	4 (2.6)	
Unemployed	5 (1.5)	2 (1.1)	3 (2.0)	

For peer review only

**Marital status**				0.21
Married	299 (92.0)	157 (90.2)	142 (94.0)	
Unmarried	26 (8.0)	17 (9.8)	9 (6.0)	
**Perceived wealth**				**0.002**
Very poor or poor	44 (13.5)	33 (19.0)	11 (7.3)	
Average	280 (86.2)	140 (80.5)	140 (92.7)	
Rich or very rich	1 (0.3)	1 (0.6)	0 (0.0)	
**Singleton/multiple pregnancy[2]**				**0.03**
Data available	261 (80.3)	144 (82.8)	117 (77.5)	
Singleton	242 (92.7)	129 (89.6)	113 (96.6)	
Multiple	19 (7.3)	15 (10.4)	4 (3.4)	
**Planned location for delivery**				**0.004**
MMH	216 (66.5)	102 (58.6)	114 (75.5)	
Other hospital	97 (29.8)	63 (36.2)	34 (22.5)	
Health centre	8 (2.5)	6 (3.4)	2 (1.3)	
Home	4 (1.2)	3 (1.7)	1 (0.7)	
**Pregnancy outcome**				**<0.001**
Data available[1]	319 (98.2)	170 (97.7)	149 (98.7)	
Ending before 20 weeks	43 (13.5)	25 (14.7)	18 (12.1)	
Live birth	191 (59.9)	69 (40.6)	122 (81.9)	
Stillbirth	81 (25.4)	73 (42.9)	8 (5.4)	
Still pregnant at discharge	4 (1.3)	3 (1.8)	1 (0.7)	

192 1) In case of missing data, the table shows the available data per category in the first row; the consequent
193 proportions are calculated with the available data as denominator
194 2) This excludes participants who were admitted with a gestational age below 20 weeks, including pregnancies
195 which ended in abortion and ectopic pregnancies
196

197 **Access to care**

198 Motivations for choosing a delivery location did not differ between MNM and controls and were
199 most often because of previous experience and expectations (36.6%) or medical indication
200 (28.0%).

201

202 *"I didn't decide yet in which hospital but I don't want to deliver at home because you can have*
203 *a problem and not be able to solve it"* – MNM participant, 25 years old, first pregnancy

204

For peer review only

The majority of MNM (59.2%) initiated their journey to MMH because they recognised that their condition was deteriorating, whilst less than a quarter (23.0%) of them said they were unaware.

"I recognized it was not good. I thought I needed to have an operation because that is what happened to my sister-in-law." – control participant, 36 years old, fourth pregnancy

Table 2 displays the various elements on access to care investigated. Over half of the MNM (55.6%) and the controls (66.1%) said to have taken more than two hours before they decided to seek medical care after starting to feel sick or labour started (p=0.18). After that, it took 36.2% of MNM and 29.1% of controls longer than 30 minutes to reach a health facility (p=0.10). Once arrived, the vast majority was attended to promptly, but some had waited longer than three hours, and MNM significantly more often than controls (9.3% vs. 5.4%, p=0.04).

Close to one fifth of all interviewees (18.6%) experienced the access to care as suboptimal, with a significant higher number of MNM than controls reporting to have encountered problems of financial, logistical or referral-related nature (22.4% MNM vs 13.9% controls, p=0.049). Many interviewees shared that they had struggled with finding and paying petrol, a borrowed car or public transportation.

"I had to wait and find a bus for six hours. There should be transport available" – MNM participant, 19 years old, second pregnancy

"I came by ambulance from [my area] and paid 10,000TSH (equivalent to 5 USD, authors). I find it not fair of them that I had to pay. What if I had no money?" – MNM participant (area of residence to MMH around a 20-minutes-drive, 8-9km), 40 years old, sixth pregnancy

For peer review only

"Because my husband was not able to buy petrol for our car, I took a dala dala (public mini bus, authors) for three hours from [my area] to MMH." – MNM participant (area of residence to MMH around one and a half hour-drive by car, 40km), 38 years old, eight pregnancy

Table 2: Experience of accessibility of care

	Total N (%)	MNM N (%)	Controls N (%)	p-value[1]
**Total**	325 (100.0)	174 (53.5)	151 (46.5)	
**Sought care X time after complaints started *(delay 1)***				0.18
Data available[2]	284 (87.4)	151 (86.8)	133 (88.1)	
Immediately	60 (21.1)	40 (26.5)	20 (15.0)	
<2 hours	35 (12.3)	19 (12.6)	16 (12.0)	
2-8 hours	96 (33.8)	45 (29.8)	51 (38.3)	
>8 hours	76 (26.8)	39 (25.8)	37 (27.8)	
Not applicable	17 (6.0)	8 (5.3)	9 (6.8)	
**Reached care X time after start of journey *(delay 2)***				0.10
Data available[2]	261 (80.3)	129 (74.1)	132 (87.4)	
<15min	70 (25.7)	36 (26.1)	34 (25.4)	
15-30min	102 (37.5)	43 (31.2)	59 (44.0)	
30-60min	49 (18.0)	24 (17.4)	25 (18.7)	
>60min	40 (14.7)	26 (18.8)	14 (10.4)	
**Received care X time after arrival in health facility *(delay 3)***				**0.04**
Data available[2]	304	158	146	
Immediately	251 (78.7)	122 (71.3)	129 (87.2)	
<3hrs	29 (9.1)	20 (11.7)	9 (6.1)	
>3hrs	24 (7.5)	16 (9.3)	8 (5.4)	
**Experienced access to care**				0.48[3]
Data available[2]	322	171	151	
Very poor	2 (0.6)	2 (1.2)	-	
Poor	10 (3.1)	5 (2.9)	5 (3.3)	
Not poor, not good	48 (14.9)	28 (16.4)	20 (13.2)	
Good	59 (18.3)	35 (20.5)	24 (15.9)	
Very good	203 (63.0)	101 (59.1)	102 (67.5)	
**Encountered problems**				**0.049[4]**
None	265 (81.5)	135 (77.6)	130 (86.1)	
Any	60 (18.5)	39 (22.4)	21 (13.9)	
Financial	30 (9.2)	24 (13.8)	6 (4.0)	
Referral from one health facility to	10 (3.1)	6 (3.4)	4 (2.6)	

For peer review only

another				
Logistics	20 (6.2)	9 (5.2)	11 (7.3)	

1) p-values are calculated with Chi-Square tests excluded the row of 'data available'; 2) the proportion of data available is calculated, the proportion of the following subcategories are calculated with the available data as the denominator; 3) this p-value has been calculated with combining the subcategories 'very poor' and 'poor'; 4) this p-value has been calculated by comparing the categories 'none' and 'any'

Quality of care

Table 3 displays the means of the outcomes per interview question reflecting quality of care. Most of these given scores are high, i.e. above four. Lowest scores regard having an informed choice (total group 2.67, MNM 2.49 and controls 2.86) and being informed (total group 3.87, MNM 3.61 and controls 4.17), including being informed about the death of their baby (total group 3.90, MNM 3.89 and controls 4.00). Mean differences between MNM and controls were statistically significant for some topics, such as having felt informed and perception of motivation of staff, but reflect small differences. One participant summarized her experience as follows:

"I am very satisfied because I am alive" – MNM participant, 28 years old, first pregnancy

Table 3: Means on a five-point scale (1=very poor, 2=poor, 3=not poor, not good, 4=good, 5=very good) and mean differences

	**Total**	**MNM**	**Controls**	
	**Mean (SD)**	**Mean (SD)**	**Mean (SD)**	**Mean difference (95% CI)**
Did you feel treated with respect?	4.54 (0.80)	4.50 (0.85)	4.58 (0.74)	-0.07 (-0.25–0.10)
Did you feel informed about treatments/ complications?	3.87 (1.54)	3.61 (1.62)	4.17 (1.40)	-0.55 (-0.89–0.22)
Did you feel you had an informed choice in the services you received?	2.67 (1.80)	2.49 (1.76)	2.86 (1.84)	-0.37 (-0.76–0.03)
Did you feel you had privacy?	4.47 (0.96)	4.36 (1.12)	4.59 (0.72)	-0.23 (-0.44–0.02)
Were you able to receive emotional support from relatives/friends?	4.47 (0.97)	4.51 (0.91)	4.42 (1.03)	0.09 (-0.12–0.30)
Did you feel you were provided with emotional support by the staff?	4.27 (1.18)	4.23 (1.20)	4.32 (1.16)	-0.09 (-0.35–0.17)
Did you feel that the staff was motivated and available?	4.41 (0.88)	4.32 (0.95)	4.52 (0.78)	-0.20 (-0.40–0.01)

For peer review only

Did you feel safe in the hospital?	4.65 (0.67)	4.63 (0.69)	4.66 (0.64)	-0.03 (-0.18–0.11)
Did you feel the health workers took the time for you?	4.51 (0.83)	4.47 (0.87)	4.56 (0.78)	-0.08 (-0.26–0.10)
Did you experience financial barriers to getting the right treatment?	4.59 (0.88)	4.47 (1.00)	4.74 (0.70)	-0.27 (-0.46–0.08)
Did you think the hospital had sufficient supplies to care for you? (Sanitation, medicine, equipment)	4.01 (0.99)	3.91 (0.97)	4.10 (1.02)	-0.17 (-0.39–0.05)
How do you feel about the way your baby was treated?	4.67 (0.77)	4.64 (0.87)	4.69 (0.70)	-0.05 (-0.28–0.18)
How do you feel about the overall experience of your admission?	4.33 (0.90)	4.24 (0.93)	4.42 (0.86)	-0.19 (-0.38–0.01)
In case baby died: how do you feel about the way you were informed on the death of your baby?	3.90 (1.54)	3.89 (1.56)	4.00 (1.48)	-0.11 (-1.08–0.85)
In case baby died: how do you feel about the way you, your baby and the baby's father were treated after your baby's death?	4.16 (1.39)	4.31 (1.26)	3.27 (1.85)	1.04 (0.16–1.91)
Would you recommend your sister/friend to deliver in the same place?	**Total N (%)**	**MNM N (%)**	**Controls N (%)**	
Yes	309 (95.1)	164 (94.3)	145 (96.0)	0.85 (0.57–1.26)
No	16 (4.9)	10 (5.7)	6 (4.0)	

As displayed in Table 4, a total of 70 interviewees (21.5%), comprising 43 MNM (24.7%), and 27 controls (17.9%), perceived the overall experience of the care during their admission as suboptimal. Most participants rated informed choice poorest, being scored as suboptimal by 57.5% of all participants. This is followed by sufficiency of supplies (28.3%), being informed in general (28.3%), and being informed in case of death of their baby (25.9%).

"If I had money I would go to a private hospital. The first doctor after the operation didn't tell me my baby died. … I felt ignored. …They didn't tell me anything. I am heartbroken here." – MNM participant, 31 years old, second pregnancy

Table 4: Suboptimal scores on quality of care reflecting absolute numbers and the proportion of the group of participants who answered with 1, 2 or 3. Odds ratios (OR) calculated for MNM vs. controls.

	**Total, N(%)**	**MNM, N(%)**	**Control, N(%)**	**OR (95% CI)**
**Total**	325 (100.0)	174 (53.5)	151 (46.5)	-
**Overall experience**	70 (21.5)	43 (24.7)	27 (17.9)	1.51 (0.88-2.59)

For peer review only

**Informed choice**	187 (57.5)	108 (62.1)	79 (52.3)	1.49 (0.96-2.32)
**Sufficient supplies**	104 (32.0)	61 (35.1)	43 (28.5)	1.36 (0.85-2.17)
**Information**	92 (28.3)	61 (35.1)	31 (20.5)	***2.09 (1.26-3.46)***
**Emtional support staff**	66 (20.3)	37 (21.3)	29 (19.2)	1.14 (0.66-1.96)
**Availability and motivation of staff**	57 (17.5)	38 (21.8)	19 (12.6)	***1.94 (1.07-3.54)***
**Time for patient**	43 (13.2)	22 (12.6)	21 (13.9)	0.90 (0.47-1.70)
**Financial barriers**	42 (12.9)	31 (17.8)	11 (7.3)	***2.76 (1.34-5.70)***
**Privacy**	41 (12.6)	29 (16.7)	12 (7.9)	***2.32 (1.14-4.72)***
**Emotional support family**	39 (12.0)	20 (11.5)	19 (12.6)	0.90 (0.46-1.76)
**Respect**	36 (11.1)	22 (12.6)	14 (9.3)	1.42 (0.70-2.88)
**Feeling of safety**	18 (5.5)	10 (5.7)	8 (5.3)	1.09 (0.42-2.84)
**Treatment of the baby**	10 (3.1)	4 (5.8)	6 (4.9)	0.57 (0.16-2.05)
		**Total N=73**	**Total N=12**	
**In case baby died: information**	22 (6.7)	20 (27.4)	2 (16.7)	***9.68 (2.22-42.11)***
**In case baby died: supportive treatment**	13 (4.0)	9 (12.3)	4 (33.3)	2.01 (0.60-6.65)

The odds of experiencing care as suboptimal were significantly higher for MNM concerning financial barriers in receiving high quality care (OR 2.76, 95% CI 1.34-5.70), privacy (OR 2.32, 95% CI 1.14-4.72), information provision (OR 2.09, 95% CI 1.26-3.46), and availability and motivation of staff (OR 1.94, 95% CI 1.07-3.54).

"They didn't tell me anything. I had no choice. I didn't feel extremely safe because some patients died. There is not enough medicine in the hospital and some check-ups needed to be done outside of the hospital, this costs a lot of money." – control participant, 23 years old, second pregnancy

"I don't know how my baby was treated. I haven't seen my baby yet at all. I don't know why, they told me I can't see him yet. I want to see him. I believe I will see him." – MNM participant, 29 years old, third pregnancy

Fig. 1 displays additional analysis on four domains, showing the proportion of participants who commented on the matter, among those who gave a high score and a suboptimal score,

For peer review only

respectively. However, less than 10% of the participants provided any comments, except on informed choice where almost 20% of them did. Participants scoring the quality as good or very good were just as likely as participants scoring quality as suboptimal to give negative comments when asked to answer freely.

Figure 1: Good and suboptimal scores on domains of information provision, having an informed choice, having privacy and feeling to have been treated with respect, and the proportions of participants in both groups who commented on the index domains.

The following quotes further exemplify how participants who gave high quantitative scores did have critical comments on those topics:

"Someone said to me: stay where you are or I will beat you, while I was in labour. When I see that doctor now, I feel very uncomfortable." - MNM participant, 30 years old, second pregnancy > scored a 4 on 'respect'

"No privacy because even delivery was open and everyone could see" – MNM participant, 31 years old, seventh pregnancy > scored a 5 on 'privacy'

"Staff should change their attitude. There should be better rules/guidelines about how to treat patients. They should treat patients with more respect and listen to us. They should be more focused." – MNM participant, 24 years old, second pregnancy > scored a 4 on 'respect'

DISCUSSION

Main findings

On their journey to facility care, women who suffered from life-threatening complications and those who did not, equally went through extensive processes preceding the decision to go to the health care facility. Around one fifth of them, MNM significantly more often so, subsequently experienced barriers, mostly due to financial constraints. Once in the hospital, care was given

For peer review only

quickly. The quality of care reported through quantitative questionnaires was experienced as high. One fifth of all participants, MNM significantly more often than controls, had an overall suboptimal experience with informed choice and availability of supplies scored lowest. Our expectation was that participants would more often comment negatively if they had given lower scores but this did not occur consistently. Generally, there was a positive quantitative assessment with critical comments expressed by a minority of the participants.

Interpretation

In our study population, women who suffered from MNM reported to have experienced financial barriers more often than the controls did. Previous work found that financial costs were a major obstacle in seeking non-emergency obstetric care, but not so much in emergency obstetric care,[14-15]. We have insufficient insights into the course of disease to conclude whether these obstacles are causally related with outcomes. Though, they might reflect how a deprived socio-economic situation creates a predisposition for poorer health outcomes. This is underscored by the fact that our study participants, who developed the near-miss complications, had completed lower levels of formal education and perceived their own wealth more often as poor. Additional research should be done to explore the nexus between economic factors and health-care seeking behaviour, like the studies from multiple countries in Sub-Saharan Africa and from Bolivia which have displayed similar difficulties with poor information provision and consequent limitations in making informed choices as experienced by our study participants,[16-20].

Our study has shown that, severe obstetric complications do not seem to significantly impact patient's perception of care, either negatively or positively. Adverse outcomes will in most cases have led to intensification of clinical care, which could partly explain a positive experience from patients who had severe complications. On the other hand, possible

For peer review only

negligence or poor communication and information provision have likely influenced perceptions negatively.

This study's methodology was mainly quantitative, but encouraged comments from the participants to enrich the numeric outcomes. Through calculations of averages we get a sense of experienced quality of care, but the valuable information for policy change really seems to come from the positive and negative extremes. The individual participants' comments reflected those extremes best, with their experiences of substandard care highlighting ample opportunities for improvement. This is most visible in their perception of quality of care which had a discrepancy between the quantitative and qualitative outcomes. We hypothesise that quantitative outcomes mainly reflect the norms in the health care system and broader society, with people adjusting their standards to what is currently available to them. Even so, qualitative data did shed a bright light on personal needs and preferences deviating from the current norm. The norm might be what it is, but that does not mean that the patient, who is depending on the health care system, finds the norm acceptable. For example, the vast majority scored 'privacy' as good or very good, whilst one in every 20 participants shared how they had to share beds, there was a lack of curtains and physical examinations were executed without shielding. The quotes reproduced in the article illustrate stories on poor treatment during hospitalization that are similar to those of other women around the world,[14-18], whose expectations of smooth access, privacy, adequate provision of information, attitude and availability of supplies reflect essential elements of high-quality maternal care as standardized by the WHO,[8]. Dissatisfaction on not meeting these expectations might not be reflected directly given the propensity to normative answers in most, but can negatively impact future service utilization and, consequently, maternal health outcomes.

Our study exemplifies the need for both quantitative and qualitative methods to get a genuine impression of patients' perspectives and experiences. Theories in the domains of social and economic welfare have argued the potential value of patients' perspectives in health research,[21], claiming how a patient's direct testimony of the experienced illness and received

For peer review only

health care might be too positive when compared to the public's supposedly more objective opinion. The concept of adaptive preferences explains how health care, which is formally perceived as substandard, might be perceived as sufficient or good by the receiving individual at that time. We argue, though, partly in line with Nussbaum's philosophy on this, that any patient's testimony, at any time and any place, is valid in and of itself,[21,22]. Furthermore, following a human rights-based approach to maternal health care, we believe it to be quintessential, in the pursuit of universal high-quality care for all, to include women and their communities in maternal health research and policy making,[23].

Strengths and limitations

This study involved a wide range and large number of patients from the maternal population in Zanzibar's main hospital, which deals with a high volume of non- and mildly complicated cases as well as with, in its role of referral hospital, the most complicated cases. Nevertheless, it is not a complete representation of the patient population, leading to selection bias. We could not include women who had not reached the hospital, including those who may suffer most from financial barriers. The same applies to the exclusion of patients who were not reachable by mobile phone, which possibly excluded those suffering more from poverty and possibly having less influence in their households and in decision-making processes regarding health. The (majority of) interviews took place in the hospital at the risk of information bias, due to hesitation to criticize the facility they were still in and, sometimes, depending on. Furthermore, we cannot exclude moderator and reporter bias, which we tried to mitigate through a well-defined methodology and interview structure.

Within the group of participants who commented critically beyond the set interview questions, we reached data saturation, thus trusting their comments to reflect a daily experienced reality and consequently providing policy makers with starting points for health care improvement.

For peer review only

Methodological challenges led to a percentage of the MNM during the study period to not have been interviewed and for a lower number of control participants to have been included. The final large number of included participants, however, makes us believe that this has not led to selection bias.

CONCLUSION

This study assessed the perception of patients on their journey to and during maternal care in Zanzibar's main health facility. First-hand testimonies, show that most women seek care in time, access MMH quickly and are treated in time. Nevertheless, a significant minority of them experiences barriers, mostly financial, in reaching care, women with a MNM more so than controls. Quality of care is valued as high and is similar for women with and without an MNM. Simultaneously, a group of participants shared more critical perceptions. There seems to be an opportunity to distinguish between normative and personal perceptions and, thus, combining quantitative and qualitative methodologies should be maintained. Further improvement of maternal health and health care will be possible when policies are based on such research, whilst being patient-centred and setting-specific.

REFERENCES

1. Alkema L, Chou D, Hogan D, et al. Global, regional, and national levels and trends in maternal mortality between 1990 and 2015, with scenario-based projections to 2030: a systematic analysis by the UN Maternal Mortality Estimation Inter-Agency Group. *Lancet* 2016;387(10017):462-74 doi: 10.1016/S0140-6736(15)00838-7.
2. Garbysch S, Nesbitt RC, Schoeps A, et al. Does facility birth reduce maternal and perinatal mortality in Brong Ahafo, Ghana? A secondary analysis using data on 119 244 pregnancies from two cluster-randomised controlled trials. *Lancet Glob Health* 2019;7:e1074–87.

For peer review only

3. World Health Organization. Quality, equity, dignity: the network to improve quality of care for maternal, newborn and child health – strategic objectives. Geneva: WHO 2018.

4. Bohren MA, Mehrtash H, Fawole B, et al. How women are treated during facility-based childbirth in four countries: a cross-sectional study with labour observations and community-based surveys. *Lancet* 2019;394:1750–1763.

5. Solnes Miltenburg A, van Pelt S, Meguid T,et al. Disrespect and abuse in maternity care: individual consequences of structural violence. *Reprod Health Matters* 2018;26(53):88-106 doi:10.1080/09688080.2018.1502023.

6. Ministry of Health, Revolutionary Government of Zanzibar. Zanzibar Annual Health Bulletin. Zanzibar, United Republic of Tanzania 2016.

7. Herklots T, van Acht L, Khamis RS, et al. Validity of WHO's near-miss approach in a high maternal mortality setting. *PLoS ONE* 2019;14(5):e0217135 doi: 10.1371/journal.pone.0217135.

8. World Health Organization. Standards for improving quality of maternal and newborn care in health facilities. Geneva: WHO 2016.

9. Fakih B, Azzah Nofly AS, Ali AO, et al. The status of maternal and newborn health care services in Zanzibar. *BMC Pregnancy and Childbirth*. 2016;16:134 doi: 10.1186/s12884-016-0928-6.

10. Gon G, Ali SM, Towriss C, et al. Unpacking the enabling factors for hand, cord and birth-surface hygiene in Zanzibar maternity units. *Health Policy Plan* 2017;32:1220-1228 doi: 10.1093/heapol/czx081.

11. World Medical Association Declaration of Helsinki: ethical principles for medical research involving human subjects. *JAMA* 2013;310(20):2191-2194 doi:10.1001/jama.2013.281053.

12. Nijagal MA, Wissig S, Stowell C, et al. Standardized outcome measures for pregnancy and childbirth, an ICHOM proposal. al. *BMC Health Services Research* 2018;18:953.

For peer review only

13. Herklots T, van Acht L, Meguid T, et al. Severe maternal morbidity in Zanzibar's referral hospital: Measuring the impact of in-hospital care. *PLoS One* 2017;12(8):e0181470 doi: 10.1371/journal.pone.0181470.

14. Norhayati MN, Surianti S, Hussain Nik Hazlina N. Metasynthesis: Experiences of women with severe maternal morbidity and their perception of the quality of health care. *PLoS One* 2015;10(7): e0130452 doi: 10.1371/journal.pone.0130452.

15. Weeks A, Lavender T, Nazziwa E, et al. Personal accounts of 'near-miss' maternal mortalities in Kampala, Uganda. *BJOG* 2005;112(9):1302-7.

16. Tunçalp Ö, Hindin MJ, Adu-Bonsaffoh K, et al. Listening to women's voices: The quality of care of women experiencing severe maternal morbidity, in Accra, Ghana. *PLoS One* 2012;7(8): e44536 doi: 10.1371/journal.pone.0044536.

17. Rööst M, Jonsson C, Liljestrand J, et al. Social differentiation and embodied dispositions: a qualitative study of maternal care-seeking behaviour for near-miss morbidity in Bolivia. *Reprod Health* 2009;29;6:13 doi: 10.1186/1742-4755-6-13.

18. Norhayati MN, Nik Hazlina NH, Ab Razak A, et al. The experiences of women with maternal near miss and their perception of quality of care in Kelantan, Malaysia: a qualitative study. *BMC Pregnancy and Childbirth* 2017;17:189 doi: 10.1186/s12884-017-1377-6.

19. Rosen HE, Lynam PF, Carr C, et al. Direct observation of respectful maternity care in five countries: a cross-sectional study of health facilities in East of Southern Africa. *BMC Pregnancy and Childbirth* 2015;15(306) doi: 10.1186/s12884-015-0728-4.

20. Mafuta EM, De Cock Buning T, Lolobi DL, et al. Factors influencing the capacity of women to voice their concerns about maternal health services in the Muanda and Bolenge Health Zones, Democratic Republic of the Congo: a multi-method study. *BMC Health Services Research* 2018;18(37) doi: 10.1186/s12913-018-2842-2.

21. Nussbaum M. Symposium on Amartya Sen's philosophy: Adaptive preferences and women's options. *Economics & Philosophy* 2001;17(1):67-88 doi: 10.1017/S0266267101000153.

For peer review only

22. Mitchell P. Adaptive Preferences, Adapted Preferences. *Mind* 2018; 127(508):1003-1025 doi: 10.1093/mind/fzy020.

23. Freedman LP. Using human rights in maternal mortality: from analysis to strategy. *Int J Gynaecol Obstet* 2001;75:51-60.

FIGURE LEGEND

Figure 1. Legend: top bar left side= suboptimal score with no comment; top bar right side= suboptimal score with comment; bottom bar left side= good score with no comment; bottom bar right side= good score with comment

SUPPLEMENTARY FILES

Table 1: Markers for organ dysfunction, identifying cases in life-threatening condition

Table 2: Interview outline

AUTHOR CONTRIBUTIONSHIP

TH, TM and BJ conceptualized the study. TH, KMS, MR and LD conducted the majority of the data collection and were responsible for data integrity. TH, SC and MPHK performed data analysis. TH and TM supervised the research on-site. BJ and AF supervised the research off-site. TH drafted the manuscript. All authors revised multiple versions of the manuscript. All authors have read and approved the final version of the manuscript.

COMPETING INTERESTS

None declared.

For peer review only

ETHICAL APPROVAL

Ethical approval was obtained from Zanzibar's Medical Research and Ethics Committee under protocol reference number ZAMREC/0002/JUN/17.

DATA SHARING STATEMENT

All data relevant to the study are included in the article or uploaded as supplementary information. Deidentified participant data is available from the study's first author (ORCID: https://orcid.org/0000-0003-4513-0063). Reuse is solely permitted after reasonable request with permission from the involved medical ethical committee, the hospital's administration and the study's principal investigator. The study protocol is available upon reasonable request.

FUNDING

No specific funding was received for this study

ACKNOWLEDGEMENTS

We thank Zuwena Ali Rashid, Vreni Bron, Suhaila Salum Yussuf, Sigrid Hilgen, Marloes Plender, Lisa Trommelen, Jenna Uijt de Haag, Jasmijn Rake, Jasmijn Broerze, Ibrahim Shaban, Elleke van der Meij, Eline Veenstra and Claire Bausch for their enthusiastic, tireless contributions to the data collection. We thank all health workers of Mnazi Mmoja Hospital's departments of Obstetrics & Gynaecology, Internal Medicine, Surgery and Intensive Care for facilitating and supporting participant inclusion and data collection. We thank the administration of Mnazi Mmoja Hospital for facilitating and supporting this study. We are deeply grateful to all the participants for opening up and sharing with us their opinions and ideas.

For peer review only

For peer review only - http://bmjopen.bmj.com/site/about/guidelines.xhtml

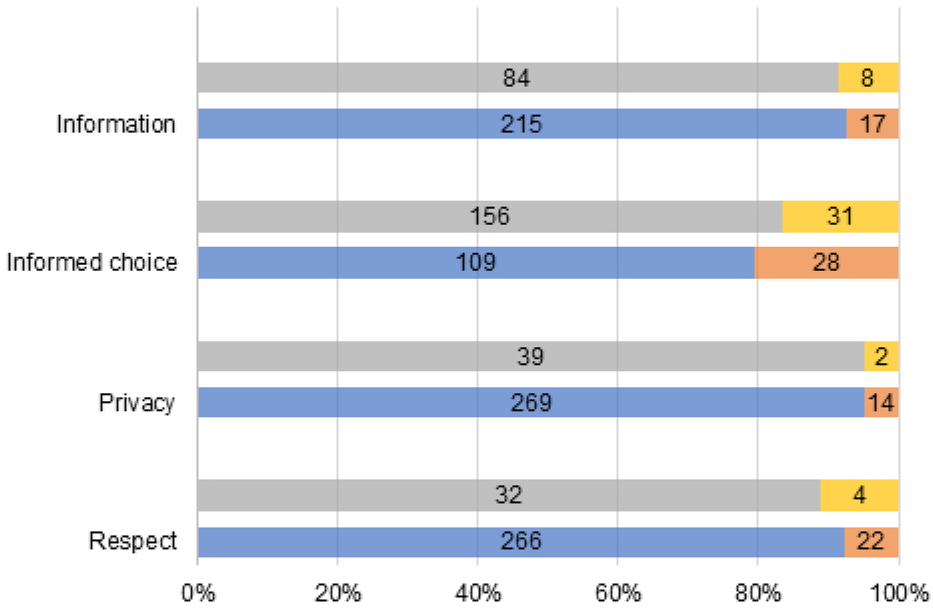

87x59mm (144 x 144 DPI)

Table 1: Markers for organ dysfunction, identifying cases in life-threatening condition

Type of organ dysfunction	Markers (clinical signs, laboratory markers and management actions)
**Cardiovascular**	Shock[1], use of continuous vasoactive drugs, cardiac arrest, cardio-pulmonary resuscitation, severe hypoperfusion (lactate >5 mmol/L or >45mg/dL)[2] or severe acidosis (pH <7.1)[2]
**Respiratory**	Acute cyanosis, gasping[3], severe tachypnea (respiratory rate >40bpm), severe bradypnea (respiratory rate <6bpm), severe hypoxemia (PAO2/FiO2 <200mmHg[2] or O2 saturation <90% for ≥60min) or intubation and ventilation not related to anaesthesia
**Renal**	Oliguria[4] non-responsive to fluids or diuretics, dialysis for acute renal failure[5] or severe acute azotaemia (creatinine ≥300umol/ml or ≥3.5mg/dL)
**Coagulation / haematological**	Clotting failure[6], use of continuous vasoactive drugs[7], massive transfusion of blood or red cells (≥5 units)[8] or severe acute thrombocytopenia (<50,000 platelets/ml)
**Hepatic**	Jaundice in the presence of pre-eclampsia[9], severe acute hyperbilirubinemia (bilirubin >100umol/L or >6.0mg/dL)
**Neurological**	Prolonged unconsciousness (lasting >12 hours)/coma[10], stroke[11], status epilepticus[12], uncontrollable fits/total paralysis
**Uterine**	Hysterectomy following haemorrhage or infection

1) Shock is a persistent severe hypotension, defined as a systolic blood pressure <90 mmHg for ≥60 minutes with a pulse rate at least 120 despite aggressive fluid replacement (>2l)
2) Laboratory test or management intervention that is not available at MMH
3) Gasping is a terminal respiratory pattern and the breath is convulsively and audibly caught
4) Oliguria is defined as a urinary output <30 ml/hr for 4 hours or <400 ml/24 hr
5) Dialysis services have been available in MMH since 26 May 2017
6) Clotting failure can be assessed by the bedside clotting test or absence of clotting from the intravenous site after 7–10 minutes
7) For instance, continuous use of any dose of dopamine, epinephrine or norepinephrine
8) In MMH extended to include all types of blood products and cases in which 5 or more units were requested but not given due to shortage
9) Pre-eclampsia is defined as the presence of hypertension associated with proteinuria. Hypertension is defined as a blood pressure of at least 140 mmHg (systolic) or at least 90 mmHg (diastolic) on at least two occasions and at least 4–6 h apart after the 20th week of gestation in women known to be normotensive beforehand. In MMH, proteinuria is defined as ≥2+ protein on dipstick.
10) Loss of consciousness is a profound alteration of mental state that involves complete or near-complete lack of responsiveness to external stimuli. It is defined as a Coma Glasgow Scale <10 (moderate or severe coma).
11) Stroke is a neurological deficit of cerebrovascular cause that persists beyond 24 hours or is interrupted by death within 24 hours
12) Condition in which the brain is in a state of continuous seizure

For peer review only

Table 2: Interview outline

PATIENT INTERVIEW – BEFORE DISCHARGE (English version)				**NO.:**					
**Topic**	**Questions**								
**1) Patient characteristics**	Age:	Level of education (primary, secondary, tertiary):							
	Gravidity/Parity:	Marital status (first-, second-, third- or fourth wife, divorced, widow, single):							
	Number of living children:	Perceived wealth:<br>Very poor – Poor – Average – Rich – Very Rich							
	Number of children of school-going age attending school:	Occupation:<br>Paid work – Self-employed – Non-paid work – Student – Housework – Unemployed for health reason – Unemployed for other reason – Other, …………………							
	Smoking?	Alcohol?	Drugs?						
**2) Family Planning**	Pregnancy planned?	If unplanned, wanted?	Did you have access to contraceptives?						
				**1**	**2**	**3**	**4**	**5**	**na**
**3) Antenatal Care (ANC)**	**How did you experience the access of ANC?** Scale: 1=impossible, 2=difficult, 3=not simple, not difficult, 4=simple, 5= very simple, na=not applicable								
	**How do you feel about your overall experience in ANC?** Scale: 1=very dissatisfied, 2=dissatisfied, 3=neither satisfied, nor dissatisfied, 4=satisfied, 5= very satisfied, na=not applicable								
	**Did you consult others for treatment during your pregnancy such as traditional birth attendants or others?**								
	a. No								
	b. Yes, traditional birth attendant								
	c. Yes, other								
	**Comments:**								
**4) Access to Health Facility**	**Where did you plan to deliver?**								
	a. Home								
	b. Health center								
	c. Other hospital								
	d. MMH								
	**What was the main motivation for delivering there?**								
	**Who accompanied you to the health facility and took care of you during hospitalization?**								
	a. Female family member								
	b. Husband								
	c. Friend								
	d. Other								
	**Who took care of your children during your hospitalization?**								
	a. Female family member								
	b. Husband								
	c. Friend								
	d. Other								

For peer review only

		1	2	3	4	5	na
	**How did you experience the access to the health facility?** Scale: 1=very poor, 2=poor, 3=not good, not poor, 4=good, 5= very good, na=not applicable						
	**If you encountered problems, what kind of problems?**						
	a. Financial						
	b. Referral						
	c. Logistic						
	d. No problems						
	**How did you come here and how long did that take?**						
	**Comments:**						
**5) Perception on Development of Disease/ Complications**	**How much time passed between when you started feeling less well and you sought medical help?**						
	**How much time passed between when you arrived in the hospital/you started feeling less well in the hospital and the moment a health worker came to see/question/examine you?**						
	**Did you recognise that you started to feel worse? If so, how did you feel and what did you think?**						
		1	2	3	4	5	na
	**Did you have knowledge about your disease/ complication(s) before you suffered from it yourself? If so, how?** Scale: 1=not at all, 2= little, 3= moderate amount, 4=good knowledge, 5=extreme good knowledge, na=not applicable						
	**Comments**						
		1	2	3	4	5	na
**6) Perceived Quality of Care during Hospitalization**	**Did you feel treated with respect?** Scale: 1=not at all, 2=a little, 3=a moderate amount, 4=very much, 5=extremely, na=not applicable						
	**Did you feel informed about treatments/ complications?** Scale: 1=not at all, 2=a little, 3=a moderate amount, 4=very much, 5=extremely, na=not applicable						
	**Did you feel you had an informed choice in the services you received?** Scale: 1=not at all, 2=a little, 3=a moderate amount, 4=very much, 5=extremely, na=not applicable						
	**Did you feel you had privacy?** Scale: 1=not at all, 2=a little, 3=a moderate amount, 4=very much, 5=extremely, na=not applicable						
	**Were you able to receive emotional support from relatives/friends?** Scale: 1=not at all, 2=a little, 3=a moderate amount, 4=very much, 5=extremely, na=not applicable						
	**Did you feel you were provided with emotional support by the staff?** Scale: 1=not at all, 2=a little, 3=a moderate amount, 4=very much, 5=extremely, na=not applicable						

For peer review only

	**Did you feel that the staff was motivated and available?** Scale: 1=not at all, 2=a little, 3=a moderate amount, 4=very much, 5=extremely, na=not applicable						
	**Did you feel safe in the hospital?** Scale: 1=not at all, 2=a little, 3=a moderate amount, 4=very much, 5=extremely, na=not applicable						
	**Did you feel the health workers took time for you?** Scale: 1=not at all, 2=a little, 3=a moderate amount, 4=very much, 5=extremely, na=not applicable						
	**Did you experience financial barriers to getting the right treatment?** Scale: 1=extremely, 2=very much, 3=a moderate amount, 4=a little, 5=not at all, na=not applicable						
	**Did you think the hospital had sufficient supplies to care for you? (Sanitation, medicine, equipment)** Scale: 1=not at all, 2=a little, 3=a moderate amount, 4=very much, 5=extremely, na=not applicable						
	**How do you feel about the way your baby was treated?** Scale: 1=very dissatisfied, 2=dissatisfied, 3=neither satisfied, nor dissatisfied, 4=satisfied, 5= very satisfied, na=not applicable						
	**How do you feel about the overall experience of your admission?** Scale: 1=very dissatisfied, 2=dissatisfied, 3=neither satisfied, nor dissatisfied, 4=satisfied, 5= very satisfied, na=not applicable						
	If your baby died:						
		**1**	**2**	**3**	**4**	**5**	**na**
	**How do you feel about the way you were informed on the death of your baby?** Scale: 1=very dissatisfied, 2=dissatisfied, 3=neither satisfied, nor dissatisfied, 4=satisfied, 5= very satisfied, na=not applicable						
	**How do you feel about the way you, your baby and the baby's father were treated after your baby's death?** Scale: 1=very dissatisfied, 2=dissatisfied, 3=neither satisfied, nor dissatisfied, 4=satisfied, 5= very satisfied, na=not applicable						
	**Comments:**						
	**SCALE** 1=an extreme impact, 2= a high impact, 3=a moderate impact, 4= little impact, 5= no impact at all, na=not applicable	**1**	**2**	**3**	**4**	**5**	**na**
**7) Perspective on Impact of Hospitalization**	**Impact on your position within your close family**						
	**Impact on your position within extended family/community**						
	**Financial and economic impact of hospitalization**						
	**Impact on educational prospects your children**						
	**Comments:**						
**8) State upon Discharge**	**Do you feel recovered at discharge?** Yes/No						
	**Do you feel able to resume your daily activities?** Yes/No						

For peer review only

**9) Recommend**	**Would you recommend your sister to deliver in the same place?** Yes/No
**10) Suggestions**	**Do you have any suggestions to improve the care for you and your baby in the future? What would you like to see different?**

For peer review only

SRQR Checklist:

S1 – title > see title, line 1-3

S2 – abstract > see abstract, page 2

Introduction

S3 – problem formulation > line 77-112

S4 – purpose or research question > line 118-122

Methods

S5 – qualitative approach and research paradigm > line 126, 148-151

S6 – researcher characteristics and reflexivity > 140-141

S7 – context > line 99-112

S8 – sampling strategy > line 127-32

S9 – ethical issues pertaining to human subjects > line 133-135

S10 – data collection methods > line 138-141

S11 – data collection instruments and technologies > line 138-141

S12 – units of study > line 141-148, line 175-178

S13 – data processing > line 152

S14 – data analysis > line 152-161, line 170-172

S15 – techniques to enhance trustworthiness > line 140-141, line 162-169

Results/findings

S16 – synthesis and interpretation > line 181-301

S17 – links to empirical data > throughout line 181-301

Discussion

S18 – integration with prior work, implications, transferability, and contribution(s) to the field > line 321-364

S19 – limitations > line 367-393

Other

S20 – conflicts of interest > line 483-484

S21 – funding > line 493-494

For peer review only
